# Stimulated Resonance Raman and Excited-State Dynamics in an Excitonically Coupled Bodipy Dimer: A Test for TD-DFT and the Polarizable Continuum Model

**DOI:** 10.1021/acs.jpca.3c02978

**Published:** 2023-08-18

**Authors:** Juan S. Sandoval, Qingbao Gong, Lijuan Jiao, David W. McCamant

**Affiliations:** † Department of Chemistry, University of Rochester, 120 Trustee Road, Rochester, New York 14627, United States; ‡ School of Chemistry and Materials Science, Anhui Normal University, Wuhu 241002, China

## Abstract

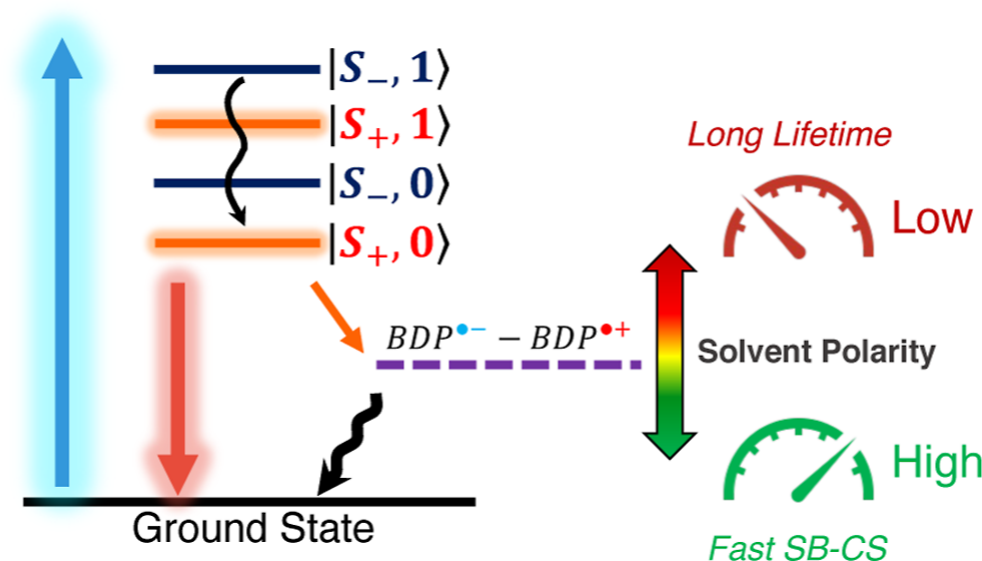

Bodipy is one of the most versatile and studied functional dyes due to its myriad applications and tunable spectral properties. One of the strategies to adjust their properties is the formation of Bodipy dimers and oligomers whose properties differ significantly from the corresponding monomer. Recently, we have developed a novel strategy for synthesizing α,α-ethylene-bridged Bodipy dimers; however, their excited-state dynamics was heretofore unknown. This work presents the ultrafast excited-state dynamics of a novel α,α-ethylene-bridge Bodipy dimer and its monomeric parent. The dimer’s steady-state absorption and fluorescence suggest a Coulombic interaction between the monomeric units’ transition dipole moments (TDMs), forming what is often termed a “J-dimer”. The excited-state properties of the dimer were studied using molecular excitonic theory and time-dependent density functional theory (TD-DFT). We chose the M06 exchange–correlation functional (XCF) based on its ability to reproduce the experimental oscillator strength and resonance Raman spectra. Ultrafast laser spectroscopy reveals symmetry-breaking charge separation (SB-CS) in the dimer in polar solvents and the subsequent population of the charge-separated ion-pair state. The charge separation rate falls into the normal regime, while the charge recombination is in the inverted regime. Conversely, in nonpolar solvents, the charge separation is thermodynamically not feasible. In contrast, the monomer’s excited-state dynamics shows no dependence on the solvent polarity. Furthermore, we found no evidence of significant structural rearrangement upon photoexcitation, regardless of the deactivation pathway. After an extensive analysis of the electronic transitions, we concluded that the solvent fluctuations in the local environment around the dimer create an asymmetry that drives and stabilizes the charge separation. This work sheds light on the charge-transfer process in this new set of molecular systems and how excited-state dynamics can be modeled by combining the experiment and theory.

## Introduction

Boron dipyrromethene and its derivatives, known as Bodipy dyes, are used in myriad applications.^1−3^ Among their properties, Bodipys are noteworthy for their small Stokes shifts, narrow absorption bands, sharp emissions, high fluorescence quantum yields, and excellent chemical and photostability.^4,5^ Furthermore, they are relatively insensitive to the polarity and pH of their environment and are reasonably stable in physiological conditions. These qualities make Bodipy promising for imaging applications,^6^ as potential sensors,^7^ as electroactive species for single-substance redox flow batteries,^8^ and as photosensitizers for applications in photodynamic therapy^9^ and photocatalysis.^10^

Different strategies to modify the structure of the boron-dipyrine core and, thus, the photophysical properties of Bodipy derivatives have been thoroughly discussed in the literature.^11−15^ One of the strategies is the formation of covalently bonded dimers such as β–β-linked dimers, meso–meso-linked dimers, and fused aromatic-linked dimers.^16,17^ Bodipy oligomers with covalent linkages have attracted much interest for their near-IR light-absorbing properties and as singlet oxygen photosensitizers and electrochemiluminescence sensors.^11,18,19^

We have previously developed a novel strategy for the controllable synthesis of ethylene-bridged Bodipy dimers based on a selective Cu(I) radical homocoupling reaction through single-electron-transfer chemistry.^20^ To our knowledge, this is the only example of ethylene-bridged Bodipys at the α position. Following this methodology, a series of α,α-ethylene-bridged Bodipy dimers were synthesized using a variety of Bodipy monomers with different substituted groups. Their steady-state photophysical properties were investigated, and the red shift and narrowing of the absorption spectrum are in line with the characteristics of J-type aggregation.^21,22^ Interestingly, a dramatic decrease in the fluorescence quantum yield with increasing polarity was observed only in the α,α-ethylene-bridged Bodipy dimers, which is not observed in their corresponding monomer, initially attributed to a possible intramolecular excimer formation upon photoexcitation.^20^ Another explanation is photoinduced symmetry-breaking charge separation (SB-CS), where a homodimer absorbs a photon and uses its energy to transfer an electron from one chromophore to the other.^23,24^ However, the excited-state dynamics of these α,α-ethylene-bridged Bodipy dimers had not been studied with time-resolved spectroscopy.

The optical properties of dimers are usually understood following Kasha’s molecular exciton theory.^22^ The photophysical properties of the aggregates arise from the Coulombic interaction between the transition dipole moments (TDMs) of neighboring molecules that splits the excited state into symmetric (+) and anti-symmetric (−) excitonic bands. When the molecular TDMs are side-by-side, H-aggregates, the coupling is positive, the higher energy (+) band is optically active, and the lower energy (−) band is forbidden. Conversely, for “head-to-tail” orientation, J-aggregates, the coupling is negative, the higher energy (−) band is optically forbidden, and the lower energy (+) band is active. As expected from the model, the H-aggregates usually show no fluorescence, while the J-aggregates are often bright.

Alternatively, the excited-state properties of π-conjugated dimers can be described using time-dependent density functional theory (TD-DFT).^25−28^ Even though DFT and TD-DFT are, in principle, exact approaches, their accuracy is mainly determined by the exchange-correlational functional (XCF).^29−31^ The choice of XCF can be crucial to make a proper interpretation of experimental data and is typically chosen based on the functional’s ability to reproduce the experimental vertical excitation energy, a single point on the excited-state potential energy surface (PES).

**Figure 1:**
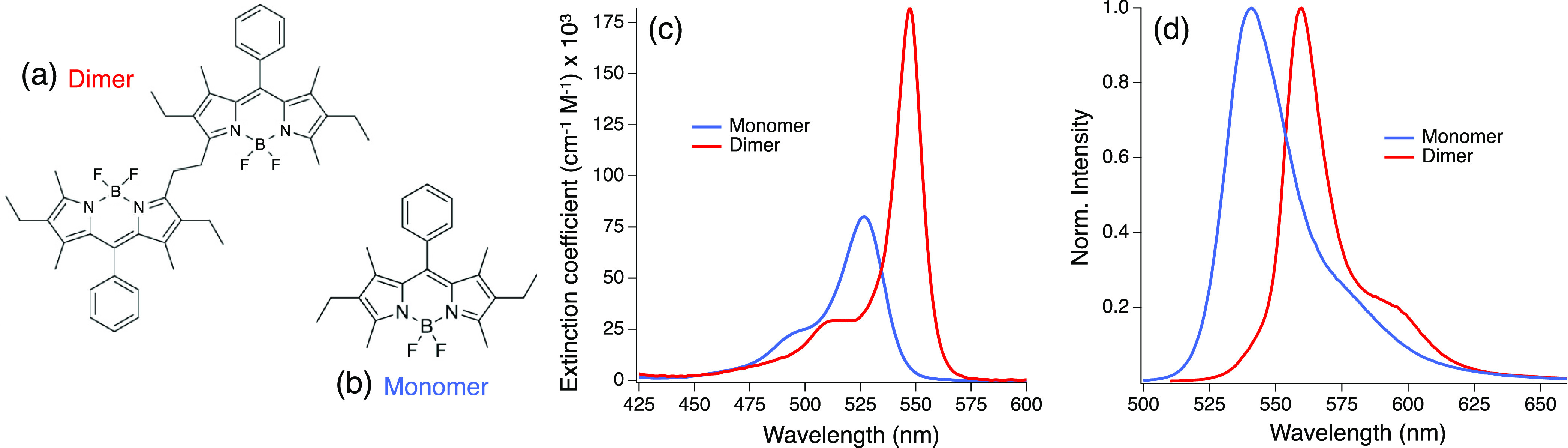
Molecular structure of (a) ethylene-bridged dimer and its corresponding (b) monomer and their absorption (c) and emission (d) spectra in benzene. The excitation wavelength was 527 nm for the monomer and 540 nm for the dimer.

In this work, we study one of these novel α,α-ethylene-bridged Bodipy dimers (Figure 1a) and its corresponding monomer (Figure 1b) using a combination of steady-state methods, ultrafast laser spectroscopy, and electronic structure calculations. Through femtosecond transient absorption (fsTA) spectroscopy, we compared the effect of solvent polarity in the excited-state dynamics in both molecular systems. Furthermore, we propose a different approach to assess the performance of different XCFs by comparing the DFT-calculated resonance Raman and oscillator strength with their experimental counterparts; this is a more stringent test that compares the energy gradient in the excited-state PES and the ground- and excited-state electronic densities,^32^ respectively. Therefore, we provide a robust criterion for choosing XCFs to study similar chromophores.

## Experimental Section

### Molar Absorptivity and Steady-State Spectroscopy

2,6-Diethyl-1,3,5,7-tetramethyl-8-phenyldipyrromethene difluoroborate was purchased from Sigma-Aldrich, and its covalent-bounded dimer (Figure 1) was synthesized using the protocol presented in Ref (20); henceforth, we refer to them as monomer and dimer, respectively. Molar absorptivities of the monomer and dimer were collected in a Shimadzu UV-1800 spectrophotometer using a 1 mm fused silica cuvette (Figure 1). Emission profiles were collected in a Spex Fluoromax-3 fluorometer (Horiba) with a photomultiplier tube detector, and the samples’ absorbances were kept below 0.1 OD. The excitation wavelengths were 527 and 540 nm for the monomer and the dimer, respectively, in a 3 mm path length optical glass cuvette (Starna). Although the excitation wavelength was within the scanned region, we did not observe any significant scattering of the excitation beam coming from the 3 mm path length optical glass cuvette.

### Electronic Structure Methods

All electronic structure calculations were performed using the Gaussian 16 software package^33^ using (time-dependent) density functional theory (TD)DFT. The visualization of molecular orbitals (MOs) was made using ChemCraft software.^34^

We chose three different exchange–correlation functionals: the popular hybrid GGA (i) BHLYP,^35^ the hybrid *meta*-GGA (ii) M06,^36^ and the long-range-corrected *meta*-GGA (iii) M11.^37^ We aimed to determine the XCF that best describes both molecules’ excited states and vibrational structure. A pre-optimized structure from B3LYP/6-311G(d) is used as an input for the ground-state geometry optimization and ground-state hessian for each functional in different solvents. For all the cases, the geometry convergence was set to *tight* and the integration grid was set to *SuperFineGrid*. To include solvent effects, we used the well-known polarizable continuum model^38^ (PCM) using two different solvents, benzene and acetonitrile (MeCN). The basis set chosen for every case is 6-311G(d). Ground-state vibrational frequencies have been scaled according to literature scaling factors: BHLYP, 0.9244;^39^ M06, 0.960;^40^ and M11, 0.958.;^40^

Vertical transition energies (*E*_v_^Abs^) and oscillator strengths (*f*_osc_) were computed with TD-DFT using the same level of theory as for the ground state. TD-DFT excited-state geometry optimization was carried out for the singlet state with the largest *f*_osc_, which is also the lowest-lying singlet excited state for all the cases in both solvents. The default geometry convergence criteria and integration grid were used only for the excited-state geometry optimization.

Resonance Raman (RR) spectra were analyzed in Gaussian16^33^ using the time-dependent framework with the vertical gradient approximation in benzene. This requires the (i) ground-state equilibrium geometry, (ii) ground-state normal mode vectors, and (iii) energy gradients (force calculation) of the excited-state potential energy surface (PES) at the ground-state geometry. The temperature was not taken into account, and the energy gradient comes from the excited state with the largest *f*_osc_. The excitation wavelength for every computed RR spectra was set to *E*_v_^Abs^ for every functional.

### Femtosecond TA Spectroscopy

A home-built non-collinear optical parametric amplifier (NOPA) pumped by a frequency-doubled output of a regenerative amplified Titanium/sapphire laser system (Spectra-Physics, 800 nm, 1 kHz) was used to produce pump pulses at 524 and 540 nm. The pulse duration was <100 fs measured by a cross-correlation obtained via the optical Kerr effect. The excitation energies were set to 40 nJ/pulse to have a good signal-to-noise ratio and avoid any two-photon events. A mechanical chopper was used to block every other pump pulse. Samples were diluted to a maximum absorbance of ∼0.7–0.8 in a 1 mm fused silica cuvette.

The white-light probe pulse was generated by focusing the 800 nm fundamental into a sapphire crystal to produce a continuum spanning from 420 to 900 nm. Before the sample, the white light spectrum was filtered using a dye solution (NIR800A, QCR Solutions Corp) to prevent residual 800 nm light from affecting the measured photochemistry and from entering the spectrograph during sample collection. After the sample, the probe was dispersed by a grating spectrograph (Acton, 300 mm fl, 150 g/mm) before reaching the charge-coupled device camera (Princeton Instruments, PIXIS 100BR). TA data was collected using perpendicular and parallel pump–probe polarizations. The parallel and perpendicular TA data were used to calculate the isotropic TA signal, Δ*A*_iso_(*t*), and the transient anisotropy, ρ(*t*).^41^

Global analysis was performed using the Glotaran software package,^42^ assuming a homogeneous, separable, and parallel kinetic model.^41^ The experimental time-resolved data *I*(λ, Δ*t*) was fit to^43,44^
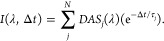
1Here, λ is the experimental wavelength variable, Δ*t* is the pump–probe time delay, τ_*j*_ is the lifetime of the *j* exponential decaying component, and DAS_*j*_(λ) is its spectral amplitude or decay-associated spectra. *N* is the number of linearly independent exponential components necessary to describe the time-resolved data. In this way, each decay-associated spectra or DAS_*j*_(λ) describes the spectral changes that occur in the time-resolved data with an associated time constant τ_*j*_.

### Femtosecond Stimulated Raman Spectroscopy

Raman pump pulses at 541 and 551 nm were generated by spectral filtering the NOPA output. First, a narrower NOPA bandwidth (∼15 nm) was achieved by adding a 1 cm cuvette of water to the white-light seed path to additionally chirp it before amplification. The output of the NOPA was spectrally filtered with a 4f filter, being dispersed by an 1800 groove/mm holographic grating and focused by a 30 cm focal length spherical lens through a ≤0.5 mm slit before being retroreflected back through the lens and grating. The resulting bandwidth was less than 20 cm^–1^, and the pulse duration was ∼1.5 ps for both wavelengths. The Raman pump was chopped at 500 Hz, and the pulse energy at the sample point was ∼20 nJ. All spectra were collected using a 2 mm path length cuvette (Starna) translated at 2 mm/s over 5 mm, and the absorption maximum of all samples was less than 0.9 OD.

The white light probe used was described for fsTA. After the amplification, the probe pulse was dispersed by a 600 groove/mm grating and focused onto the CCD camera. The scanning multichannel technique (SMT) was utilized to increase the signal-to-noise ratio by eliminating the systematic noise pattern.^45^ Specifically, the Raman signal is shifted by one pixel 20 times and then averaged for 2 s. This process is repeated 10 times for a total time of 6.67 min. The Raman shift axis was calibrated to the cyclohexane standard.

## Results and Discussion

### Absorption and Emission Spectroscopy

The absorption and emission spectra of the Bodipy monomer and dimer in benzene are shown in Figure 1c. Precise calculation of *E*_00_^Abs^ and the extinction coefficient are shown in the Supporting Information (Figures S1–S3). The Bodipy monomer shows a maximum absorption λ_max_^Abs^ at 526 nm (ε_*526nm*_ = 80, 660 *M*^–1^ cm^–1^, fwhm = 835 cm^–1^), the *S*_0_(*v* = 0) → *S*_1_(*v* = 0) transition, henceforth “0–0”. The “0–1” transition appears at 498 nm, 1105 cm^–1^ from the “0–0” transition. The dimer shows an λ_max_^Abs^ at 548 nm (ε_*548nm*_ = 180, 340 *M*^–1^ cm^–1^, fwhm = 502 cm^–1^), red-shifted by 711 cm^–1^ from the monomer peak.

Figure 1d shows the emission spectra of monomer and dimer excited at 527 and 540 nm, respectively. The monomer (dimer) emission maximum λ_max_^Em^ appears at 541 (560) nm, which corresponds to a Stokes shift of 500 (408) cm^–1^. The fwhm of the emission peaks are 979 and 572 cm^–1^ for the monomer and dimer, respectively. The dimer’s narrower red-shifted absorption and emission spectra are consistent with the characteristics of a J-aggregate.^20−22^ The monomer’s TDM is parallel to the long axis of the molecule, as shown in Figure S4. Additionally, we present the spatial orientation of the TDM on the structure of the dimer (Figure S5), which confirms the assignation of J-aggregate.

With steady-state spectroscopy is possible to calculate two experimental observables that can be used to benchmark the performance of XCF functionals to describe excited states, (i) the “0–0” energy gap (*E*_00_^Abs^) and (ii) the oscillator strength (*f*_osc_). We define *E*_00_^Abs^ as the energy corresponding to λ_max_^Abs^, and the experimental oscillator strengths are calculated by integrating the absorption spectra in the usual manner.^46^ Note that we are using *E*_00_^Abs^ to compare with the TD-DFT results because the DFT incorporates the effect of the solvent stabilization of the ground-state molecule via the PCM. If TD-DFT was performed under vacuum, then it would be more appropriate to compare it with the traditional *E*_00_, defined as the point of intersection between the normalized absorption and emission spectra. Tables 1 and 2 show the *f*_osc_ and *E*_00_^Abs^ for the monomer and the dimer. From Kasha’s exciton coupling theory,^22^ we expect the dimer to have an oscillator strength twice the monomer’s if the two monomeric TDMs are parallel in the dimer; however, the increment is around 53% relative to the monomer’s.

**Table 1:** Experimental and DFT-Calculated Oscillator Strength (*f*_osc_) and *E*_00_^Abs^ for the Bodipy Monomer Lowest Energy Transition (*S*_0_ → *S*_1_) in Benzene

functional	*f*_osc_	error % *f_osc_*	*E_v_* [cm^–1^]	*RE_I_* [cm^–1^]	*E*_00_^Abs^ [cm^–1^]	error % *E*_00_^Abs^
BHLYP	0.778	18.1	23,036	285.6	22,750	19.7
M06	0.696	5.6	22,148	271.2	21,877	15.1
M11	0.758	15.1	22,036	297.7	21,738	14.3
**experiment**	0.646			500^a^	19,011	

a Stokes shift obtained from the absorption and emission spectra.

**Table 2:** Experimental and DFT Calculated *f*_osc_ and *E*_00_^Abs^ for the Two Lowest Energy Transitions for the Bodipy Dimer in Benzene; (1) *S*_0_ → *S*_1_ and (2) *S*_0_ → *S*_2_

functional	*f*_osc_^(1)^	error % *f*_osc_^(1)^	*E*_00_^Abs(1)^ [cm^–1^]	error % *E*_00_^Abs(1)^	*f*_osc_^(2)^	*E*_00_^Abs(2)^ [cm^–1^]
BHLYP	1.571	60.7	22,081	21.0	0.0	23,254
M06	1.153	17.9	20,556	12.6	0.0	22,246
M11	1.553	58.8	21,200	16.2	0.0	22,271
**experiment**	0.990		18,248			

### Femtosecond Stimulated Raman Spectroscopy

**Figure 2:**
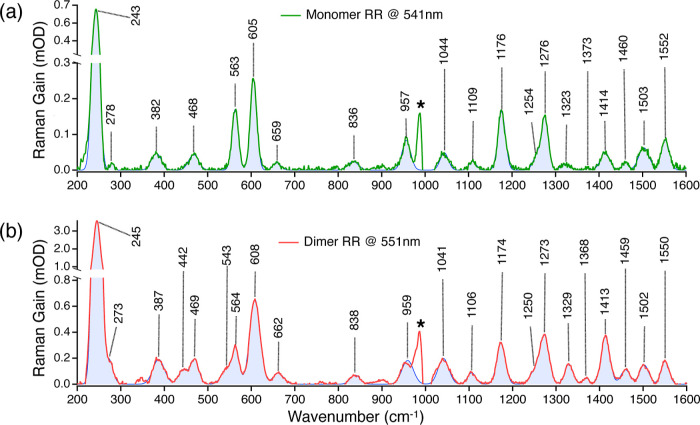
Resonance Raman spectra and their fit for the Bodipy (a) monomer and its (b) dimer at 541 and 551 nm, respectively. The asterisk marks a residual feature from solvent subtraction.

Femtosecond stimulated Raman spectroscopy (FSRS) allows the acquisition of ground-state RR spectra free of fluorescent background.^47,48^ The ground-state Raman spectra were collected at two different excitation wavelengths, 541 nm (18,484 cm^–1^) and 551 nm (18,149 cm^–1^). Figure 2 presents the resonance Raman (RR) spectra of the monomer (a) and the dimer (b) in benzene. The assignment and a complete description of the normal modes coupled with the electronic transition for the monomer can be found in Figure S12. From (b), we argue that the modes present in the dimer are the same modes identified in the monomer, consistent with prior Raman studies of TDM-coupled aggregates.^49^ However, there are two additional modes, at 442 and 543 cm^–1^, in the dimer associated with torsion in the ethylene bridge.

### Resonance Raman Intensities by DFT/TD-DFT

**Figure 3:**
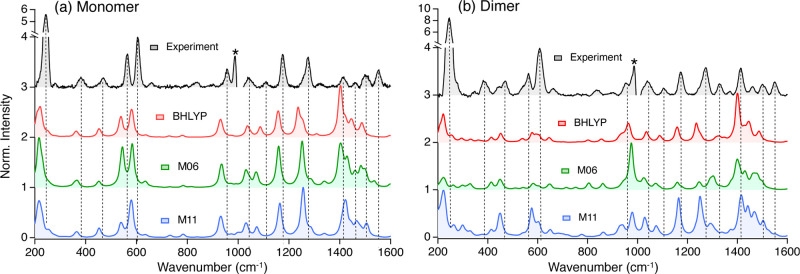
Comparison between the DFT-calculated and experimental resonance Raman spectra. Below the experimental spectrum are the DFT spectra calculated using the labeled functional for (a) the monomer and (b) its dimer. The experimental RR spectra were normalized with respect to the 605 and 608 cm^–1^ peak for the monomer and dimer, respectively. The asterisk marks a residual feature from solvent subtraction.

Here, we propose to compare the accuracy of different XCFs by their capability to reproduce the experimental RR spectra qualitatively. Figure 3 compares the RR spectra obtained through different XCFs with the experimental RR spectra for the (a) monomer and its (b) dimer. Panel (a) shows a good agreement between the calculated and observed monomer spectra for each functional. In the low-frequency region, all XCFs accurately identify all the modes observed in the experiment; however, their relative intensities are not properly described, particularly the intense band observed at 243 cm^–1^. The modes are identified correctly in the mid-frequency region, 500–1300 cm^–1^, and their relative intensities closely match the experiment. However, in the region between 1300 and 1600 cm^–1^, none of the functionals adequately identify the peaks and their intensities.

Figure 3b compares the DFT-calculated and experimental RR spectra for the dimer. Unlike the monomer, the DFT-calculated RR spectra are inconsistent with each other, and their ability to reproduce the experimental spectrum is inferior. The discrepancies between DFT-calculated and observed dimer spectra are most noticeable in the low- and mid-frequency regions. In the high-frequency region (above 1000 cm^–1^), the calculated dimer spectra resemble the calculated monomer spectra, with similar discrepancies with the experiment.

A possible explanation for the discrepancy between the experimental and the TD-DFT- calculated dimer’s RR spectrum, regardless of the accurate description of the monomer, comes from the potential energy surface (PES) associated with exciton-coupled dimers and the assumptions in the standard computation of the resonance Raman excitation profiles. Models to describe excited-state PES of two electronically coupled dimers^50−55^ and methods to compute RR line shapes^56−58^ have been thoroughly discussed in the literature and can be found elsewhere. The monomer appears to be well described by the standard model in which the excited-state nuclear PES is described as displaced harmonic oscillators along each normal mode coordinate. The excited-state PESs of the exciton-coupled dimer are dramatically affected by the excitonic coupling and the displacement between monomeric ground- and excited-state PESs (Δ), leading to highly nonharmonic surfaces in coupled dimers.^53,54^ At the same time, the assumptions to compute RR and absorption spectra rely on the harmonicity of the excited-state PES.^56,58^ Thus, the discrepancies in the dimer’s calculated RR spectrum are associated not with the accuracy of different XCFs to describe the excited-state PES but with the assumptions to calculate RR line shapes. We will address this inquiry in detail in our next work.

The experimental RR spectra allow us to assess the performance of different XCFs and TD-DFT to describe excited-state PES in the Frank–Condon region. The monomer’s RR spectrum can be qualitatively described by any of the three XCFs employed. Thus, we have confidence in the TD-DFT-calculated displacements (Δ) and reorganization energy, values that are used to calculate the monomeric *E*_00_^Abs^. Conversely, the oscillator strength is the main criterion to benchmark the XCF performance to describe the dimer’s excited state since its prediction of the RR spectrum is inaccurate.

### Electronic Structure Calculations

To obtain *E*_00_^Abs^ from DFT/TD-DFT, it is necessary to compute the vibrational frequencies and the Hessian matrix. Then, within the independent mode displaced harmonic oscillator model (IMDHO) and assuming that the ground- and excited-state PESs have the same frequency, *E*_00_^Abs^ can be computed as follows:
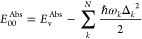
2where the second term on the right-hand side of the equation is the internal reorganization energy (RE_*i*_), *E*_v_^Abs^ is the vertical absorption energy (direct output from TD-DFT calculations), *N* is the total number of normal modes, and ω_*k*_ and Δ_*k*_ are the frequency and unitless displacement of mode *k*, respectively. *E*_v_^Abs^, ω_*k*_, and Δ_*k*_ are directly computed from DFT/TD-DFT.

Table 1 compares the TD-DFT and experimental *E*_00_^Abs^ and *f*_osc_ for the lowest-lying energy transition in the monomer. *E*_00_^Abs^ for M11 and M06 is ∼3000 cm^–1^ higher than the experimental value, consistent with previous results;^59,60^ for BHLYP, the error is ∼4000 cm^–1^, higher than previously reported.^60,61^ Higher energy transitions showed a negligible oscillator strength, *f*_osc_ ≤ 0.1 (Table S1). The relative error for *E*_00_^Abs^ and *f*_osc_ is also presented, and M06 performs better than the other functionals.

Table 2 presents *E*_00_^Abs^ and the oscillator strengths for the dimer from both TD-DFT and the experiment. The oscillator strength is directly related to the TDM, and a method that accurately describes the oscillator strength and the TDM should also correctly describe the ground- and excited-state electronic densities.^32^ The oscillator strength of the dimer obtained with M11 and BHLYP is roughly twice the monomer’s oscillator strength (*f* _osc_^Dimer^ ∼ 2*f* _osc_^Mono^), as expected from the exciton coupled theory^22^ if the TDMs of the two monomers are parallel. However, the experimental spectra (Figures S2 and S3) indicate that the dimer’s oscillator strength increases only by 53% relative to the monomer. The oscillator strength calculated with M06 XCF for both molecules closely matches the experimental results; therefore, it better describes the ground- and excited-state electronic densities and the TDM. Henceforth, we use the M06 XCF to investigate the monomer and dimer properties using DFT/TD-DFT.

**Figure 4:**
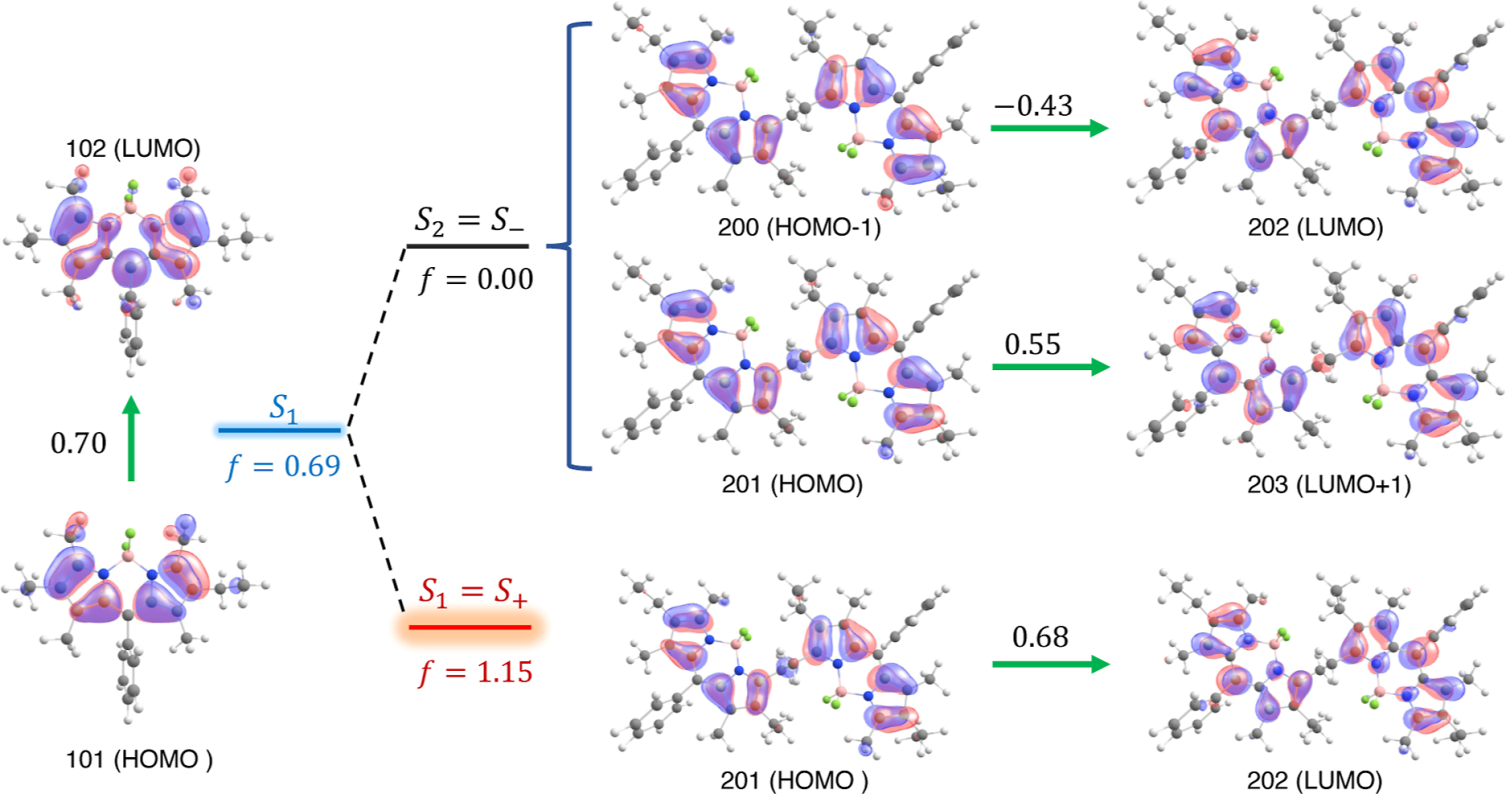
*S*_0_ → *S*_1_ electronic transitions for the monomer and first two lowest energy transitions for the dimer, *S*_0_ → *S*_1_ and *S*_0_ → *S*_2_. The Kohn–Sham molecular orbitals involved in the transition, oscillator strength, and orbital contribution are presented. *S*_+_ and *S*_–_ correspond to lower and upper exciton states, i.e., the symmetric and antisymmetric linear combination of the monomeric *S*_1_ states: , where A and B are different monomeric subunits.

The first five electronic transitions of the monomer in benzene were calculated, and the oscillator strength, orbital contribution, and energy for each transition *E*_v_^Abs^ can be found in Table S1. The DFT Kohn–Sham orbitals involved in the transition are presented in Figure S8. As shown in Figure 4, the lowest energy transition (*S*_0_ → *S*_1_) at 22,148 cm^–1^ arises predominantly from a transition from the highest occupied molecular orbital (*H*, orbital 101) to the lowest unoccupied molecular orbital (*L*, orbital 102). The second transition (*S*_0_ → *S*_2_) at 27,642 cm^–1^ arises mostly from *H* – 1 to *L*. The first electronic transition has, by far, the largest oscillator strength compared with the next five transitions, and thus, we conclude that this transition is responsible for the absorption of light in the visible region.

The first 10 electronic transitions were calculated for the dimer in benzene using the M06 functional (Table S3). The DFT Kohn–Sham orbitals involved in the first five transitions are presented in Figure 4 and Table S3. The lowest energy transition (*S*_0_ → *S*_1_) has a large oscillator strength and is dominated by a single *H*(orbital 201) → *L*(orbital 201) transition at 20,781 cm^–1^, in which both *H* and *L* are symmetrically shared across both monomer sites in the dimer. The transition with the second lowest energy (*S*_0_ → *S*_2_) at 22,472 cm^–1^ is dark (*f*_osc_ = 0.00) and is composed by an even contribution from the (*H* – 1) → *L* and *H* → (*L* + 1) transitions. The oscillator strength associated with the lowest energy transition is 1.153, the largest *f*_osc_ among the first 10 transitions (Table S3), and responsible for the absorption of light in the visible region.

In Figure 4, we show that the electronic transitions in the dimer are intrinsically related to the *S*_0_ → *S*_1_ of the monomer. The (*H* – 1), *H*, *L*, and (*L* + 1) used to construct the *S*_0_ → *S*_*i*_ (*i* = 1, 2) dimer transitions are symmetric and antisymmetric combinations of the monomers’ molecular orbitals originated from two *H* and two *L*. A similar observation was reported previously.^28^ Note that the monomer’s *S*_1_ and *S*_2_ are well separated (0.7 eV), so that a strong mixing of *S*_2_ and *S*_1_ as a result of the *S*_1_ proximity can be excluded.^28^ The first vertical transition in the dimer, *S*_0_ → *S*_1_, is strongly allowed (*f*_osc_ = 1.15), and the increase of the dimer *f*_osc_ relative to the monomer is 65%. The second vertical transition *S*_0_ → *S*_2_ is forbidden (*f*_osc_ = 0.00), regardless of the functional used (Table S3). This TD-DFT description of the dimer excited states is consistent with Kasha’s dimer model^21,22^ that describes the optical properties of two covalently bounded chromophores. As described above, the first two lowest energy transitions can be understood in terms of the monomer lowest energy transition and the simple exciton coupled theory, and the *S*_1_ and *S*_2_ dimer states can be associated with the symmetric (*S*_+_) and antisymmetric (*S*_–_) linear combination of the monomeric *S*_1_ states.

### Excited-State Dynamics

**Figure 5:**
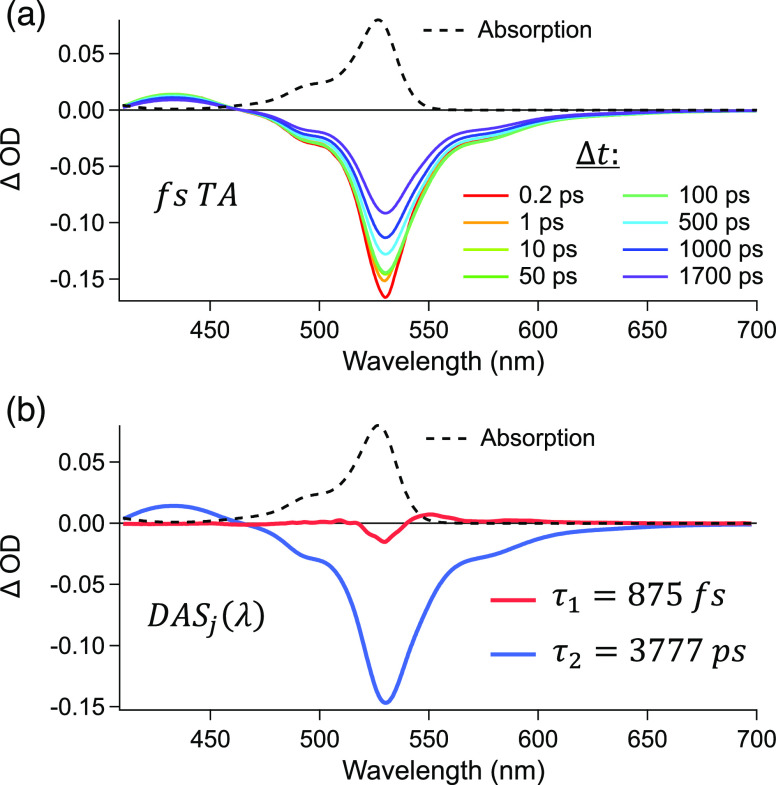
(a) fsTA at different time delays (Δ*t*) and (b) DAS_*j*_(λ) spectra of the monomer in benzene excited at 524 nm. The absorption spectrum is shown in black dashed lines.

**Figure 6:**
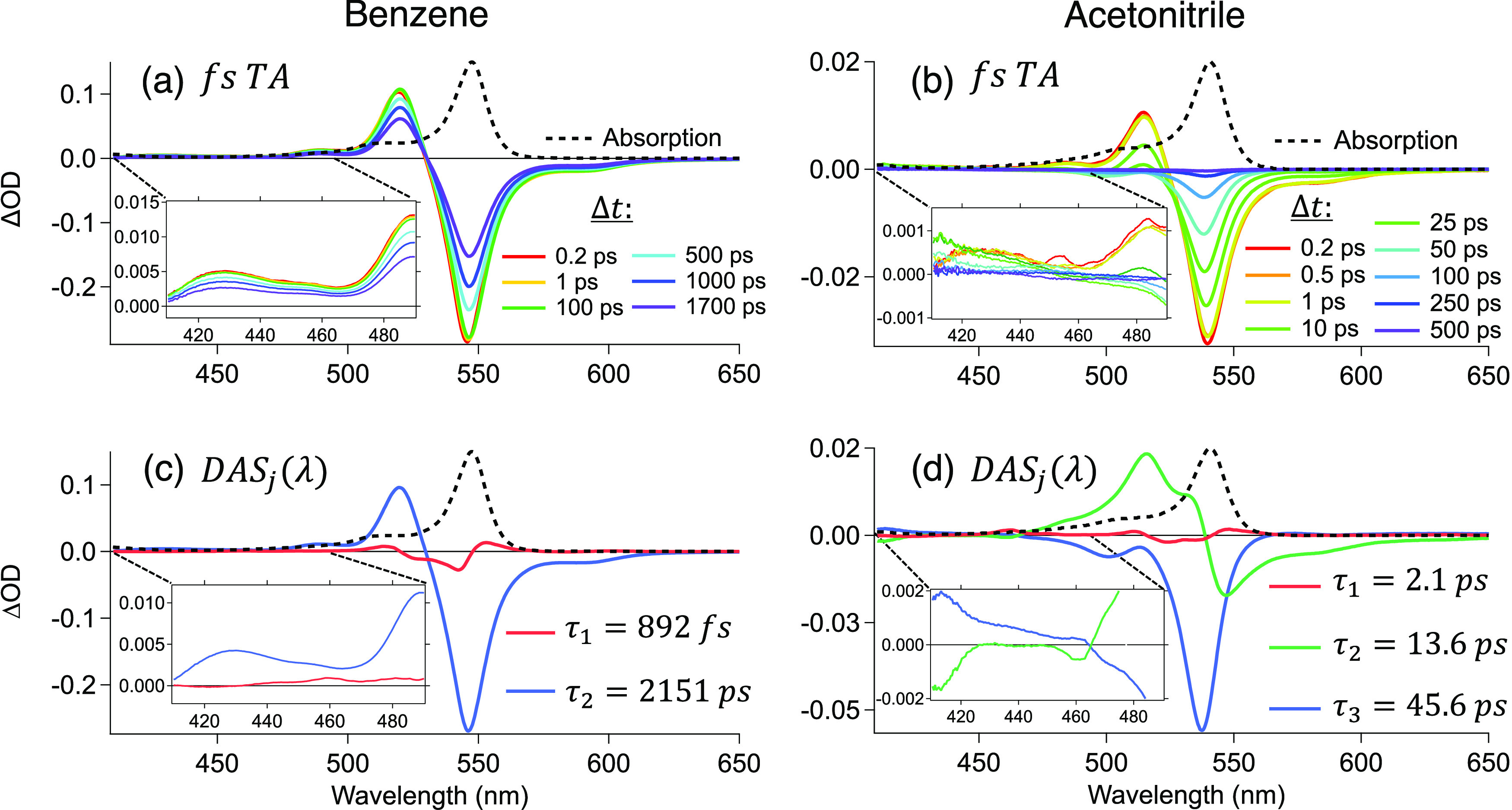
fsTA spectra at different time delays (Δ*t*) and DAS_*j*_(λ) of dimer in benzene (a,c) and acetonitrile (b,d) excited at 540 nm. The absorption spectra are presented in black dashed lines. Insets are a zoomed-in view of the 410–490 nm region.

The solvent-dependent excited-state dynamics of the monomer and dimer were determined via fsTA. The fsTA spectra of the monomer, pumped at 524 nm, and dimer, pumped at 540 nm, are presented in Figures 5 and 6, respectively. After photoexcitation, the monomer fsTA spectrum, Figure 5a, exhibits a weak positive excited-state absorption (ESA) from ∼410 to 460 nm, alongside a negative ground-state bleach (GSB) that mirrors the ground-state absorption between 460 and 540 nm. Stimulated emission (SE) bands appear on the red side of the GSB, between 540 and 680 nm. The same behavior is observed in both benzene and acetonitrile (Figure S6).

The analysis and interpretation of fsTA were accomplished using global analysis. For the monomer, regardless of the solvent, two DAS(λ) components are found (Figure 5b). The first (red) is an 875 fs component associated with the excited-state reorganization and fluorescence Stokes shift. The subsequent DAS(λ) (blue) is assigned to the singlet excited-state lifetime of 3777 ps due to the simultaneous decay of ESA, GSB, and SE. In MeCN, the same behavior is observed, though with a 4106 ps lifetime.

In Figure 6(a) and (b), we present the fsTA spectrum of the dimer in different solvents after photoexcitation with a 540 nm light. In benzene (Figure 6a), a negative GSB and SE are observed from 530 to 630 nm, and a relatively sharp ESA feature appears between 460 and 530 nm. The spectrum decays evenly over the 2 ns of our delay stage. In contrast, the excited-state dynamics in MeCN are drastically different. In Figure 6b, we observe the initial ESA, GSB, and SE in similar regions than in benzene; however, there is a rapid loss of SE and ESA in the first 15 ps and then a recovery of the ground state by 500 ps. In the 410–490 nm region, we observed a new weak ESA after 10 ps that later decays evenly to zero (Figures 6b inset). Conversely, we observe a smooth decay of the initial ESA in Figure 6a inset.

Figure 6c shows the DAS(λ) from global fitting with the corresponding time constants for the dimer in benzene. The first component (red) is associated with excited-state and solvent reorganization with a time constant of 892 fs, and the second component (blue) corresponds to the lowest excitonic-state lifetime of 2151 ps. In MeCN (Figure 6d), the first component (red) is assigned to an ultrafast reorganization with a time constant of 2.1 ps. The next component (green) exhibits a loss in ESA at 470 and 535 nm (positive DAS(λ) amplitude), a complete loss of SE above 540 nm (negative DAS(λ) amplitude), and the growth of new ESA bands (negative DAS(λ)) between 410 and 470 nm (Figure 6d inset) with a time constant of 13.6 ps, indicating the population of a non-emissive transient state different from what was observed in benzene. This can be associated with forming a charge-separated state (CSS), in which the positive band at ∼420 nm can be attributed to the Bodipy cation and anion species observed in prior studies.^24,62−65^ This is also supported by the drastic change in the dimer’s fluorescence quantum yield, which is 0.99 in toluene and 0.01 in acetonitrile.^20^ Finally, the 45.6 ps component (blue) mirrors the ground-state absorption spectra (fwhm = 15.5 nm) and corresponds to the ground-state recovery (fwhm = 15.6 nm). Similar results were obtained in MeOH, Figure S7, where SB-CS occurs in 29.6 ps and the lifetime of CSS is 202 ps. These results suggest a loss in the population of the lowest excitonic state in the first 13.6 ps via SB-CS, forming a CSS with a lifetime of 45.6 ps.

**Figure 7:**
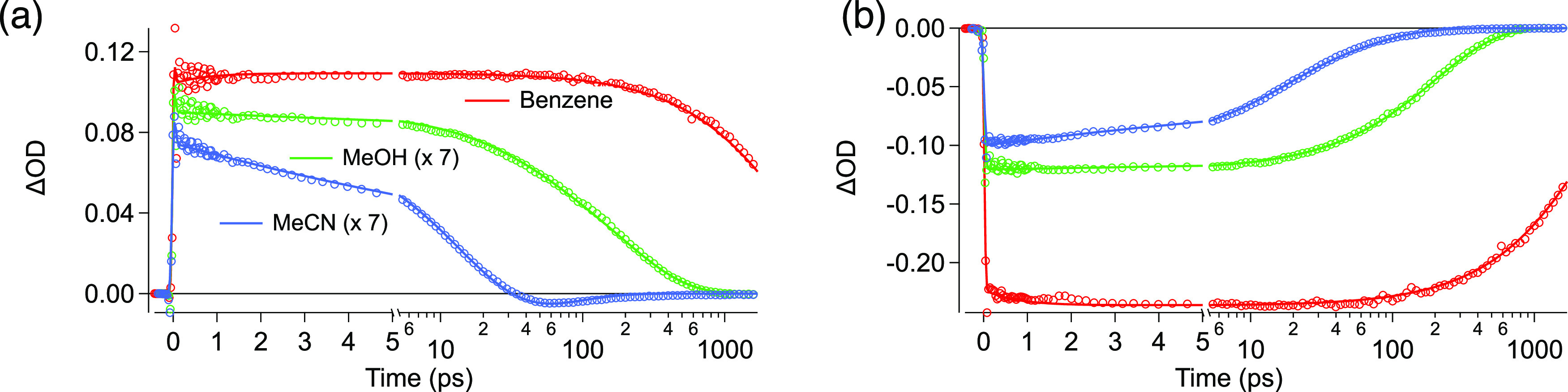
Single-wavelength kinetic traces of the Bodipy dimer in benzene, methanol (x 7), and acetonitrile (x 7) at two different wavelengths: (a) 515 and (b) 550 nm.

Figure 7 shows the resulting kinetics traces from the dimer’s global analysis at two different regions in three different solvents. In (a), we observed a slight loss of amplitude in the ESA region after photoexcitation (∼1 ps), assigned to an ultrafast excited-state reorganization. Then, the amplitude steadily decreases in polar solvents, being more pronounced in MeCN, where there is a complete loss of ESA after 40 ps. In (b), the kinetic traces show the ground-state recovery in different solvents where a similar behavior is observed, with faster recovery with increasing solvent polarity.

The feasibility of the photoinduced electron transfer and formation of a CSS is determined by the energy gap between the optical allowed state and the ion pair, calculable using the Rehm–Weller equation.^66^ The hard-sphere radii of the donor and acceptor are taken as half of the donor–acceptor distance. The details of cyclic voltammetry measuring the oxidation and reduction potentials of the monomer can be found in the Supporting Information, Figure S10. As a result, the Rehm–Weller formalism places the CSS energy in benzene, MeOH, and MeCN at 3.75, 1.85, and 1.84 eV above the ground state of the dimer. Compared to the dimer *E*_00_^Abs^, the estimated free energy of charge separation (Δ*G*_CS_) gives +1.8, −0.08, and −0.09 eV in benzene, MeOH, and MeCN, respectively, making feasible the formation of a CSS in polar solvents and infeasible in nonpolar solvents after photoexcitation. (Note that the free energy in benzene is higher than expected, possibly due to the overestimation of the polarity of nonpolar solvents in the Born dielectric continuum model,^67,68^ as previously reported).

SB-CS can be studied within the Marcus rate theory for electron transfer.^69,70^ The Marcus parameters (total reorganization energy λ_CS_^T^, the activation energy Δ*G*_CS_^#^, electronic coupling *V*_CS_, and time constant τ_CS_) for charge separation in different solvents are presented in Table 3. To calculate *V*_CS_, we used the observed time constant for charge separation (from fsTA) in MeOH and MeCN and the classical Marcus rate equation (details provided in the Supporting Information). The total reorganization energy λ_CS_^*T*^ is formed by the solvent shell reorganization energy (λ_CS_^S^) and solute molecular structure reorganization energy upon charge separation (λ_CS_^*i*,4p^). The former is calculated using the dielectric continuum theory,^70^ and the latter is calculated within the four-point approximation^71^ (details found in the Supporting Information). The activation energy Δ*G*_CS_^#^ is defined as follows^69^
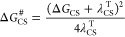
3

**Table 3:** Marcus Rate Parameters for Charge Separation^a^

solvent	Δ*G*_CS_ (eV)	λ_CS_^T^ (eV)	λ_CS_^S^ (eV)	λ_CS_^*i*,4p^ (eV)^b^	Δ*G*_CS_^#^ (eV)	τ_CS_ (ps)^c^	*V*_CS_ (eV)
Benzene	1.80	0.31	0.02	0.29	3.57		
MeOH	–0.08	1.10	0.83	0.27	0.24	29.6	0.15
MeCN	–0.09	1.09	0.82	0.27	0.23	13.6	0.18

a Driving force Δ*G*_CS_, total reorganization energy λ_CS_^T^, solvent reorganization energy λ_CS_^S^, inner sphere reorganization energy λ_CS_^*i*,4*p*^, activation energy Δ*G*_CS_^#^, electronic coupling *V*_CS_, and time constant τ_CS_.

b The inner sphere reorganization energy is calculated using the four-point method.

c Obtained from fsTA and global analysis.

While the negative free energy (Δ*G*_CS_ < 0) showed that electron transfer is a thermodynamically allowed process, the strong effect of solvent reorganization energy (λ_CS_^*S*^) on the kinetic barrier (Δ*G*_CS_^#^) explains the picosecond timescale for charge separation in polar solvents (Table 3), which is consistent with our observations of a faster rate of electron transfer as we increased the polarity.

Conversely, the driving force for charge recombination (CR) decreases as the solvent polarity increases. The free energy for CR in MeCN (Δ*G*_CR_ = −2.23 eV) is less negative than in MeOH (Δ*G*_CR_ = −2.25 eV), and the total reorganization energy in MeCN (λ_CR_^*T*^ = 1.08) and MeOH (λ_CR_^*T*^ = 1.07) places the CR in the inverted regime (−Δ*G*_CR_ > λ_CR_^*T*^). The classical equation for electron-transfer rate is no longer valid in this regime because it completely ignores nuclear tunneling.^23^ However, as expected in the inverted regime, the CR rates are faster in higher polarity solvents. Therefore, the Marcus theory of electron transfer provides a theoretical framework consistent with our experimental observations.

Covalently linked Bodipy dimers that undergo SB-CS have been previously reported.^24,63,64,72−74^ In these dimers, the *meso* or β position of the chromophores can be directly linked through a C–C bond or bridged by a spacer. Due to the orthogonal or near-orthogonal configuration achieved with this linkage type, the dimer’s absorption spectrum resembles the individual chromophores, indicating negligible or minimal excitonic coupling. Minor structural changes can significantly affect the electron transfer and charge recombination ratio,^24^ making it difficult to compare with our results. Here, for the first time, we present the SB-CS of an α,α-ethylene bridge Bodipy dimer^20^ exhibiting clear J-type aggregation characteristics.^21,22^ Other examples of molecular dimers in which the photophysical properties of molecular systems exhibiting SB-CS have been extensively studied previously, and our results are consistent with that prior work.^68,74−81^

After exploring the thermodynamics and kinetics for charge separation and recombination, we now analyze the structure rearrangement after photoexcitation and the electronic states involved in the excite-state dynamics. To characterize the nature of the electronic transition, we calculated the natural transition orbitals (NTOs), which provide a compact and simpler orbital representation of the one-electron transitions in terms of a hole (where the electron is before excitation) and a particle (where the electron is after excitation).^82^

We performed *S*_1_ geometry optimization of the monomer in benzene using M06 XCF. We found minor changes between the ground and *S*_1_ optimized structures (Figures S13 and S14); the only evident change occurs along the dihedral angle between the boron-dypyrine core and the phenylene aryl group, from 83.1*°* in the ground state to 70.7*°* in the *S*_1_ state. This is consistent with the monomer’s global analysis, which shows a very weak DAS(λ) component (Figure 5) associated with ultrafast reorganization. Figure S13 shows the NTOs associated with the first two lowest energy transitions for the monomer at the ground-state geometry. From the figure, it is clear that *S*_0_ → *S*_1_ and *S*_0_ → *S*_2_ are local excitations, and a single set of NTOs entirely describes them. At the *S*_1_ geometry (Figure S14), the two lowest energy transitions are also localized. The oscillator strength at the *S*_0_ minimum is *f*_osc_ = 0.696, while at the *S*_1_ minimum decreases to *f*_osc_ = 0.619; however, *S*_0_ → *S*_1_ remains the strongest transition. Thus, the ∼4 ns component observed in the fsTA corresponds to the monomer’s *S*_1_ lifetime.

The dimer’s electronic transition responsible for the absorption (*S*_0_ → *S*_1_) was characterized before in Figure 4 and can be understood within the exciton-coupled theory^22^ formalism. We performed TD-DFT excited-state geometry optimization using the M06 XCF on the dimer’s *S*_1_ state in benzene. Similar to the monomer in benzene, the structural differences relative to the ground-state geometry are negligible (Figure S11e), which is consistent with the weak DAS(λ) component associated with ultrafast reorganization (Figure 6c,d). At the *S*_1_ minimum, there is a slight decrease in the oscillator strength (*f*_osc_ = 1.12) of the *S*_0_ → *S*_1_ transition and subtle changes in the orbital composition (Table S3). The NTOs (Figure S15) shows that the lowest energy transition is delocalized among both chromophores at the *S*_1_ minimum, which aligns with exciton-coupled theory.^22^ We conclude that the ∼2 ns component observed in benzene corresponds to the lifetime of the *S*_1_ state.

In an attempt to identify the energy and electronic structure of the CSS, we performed TD-DFT excited-state geometry optimization on the dimer *S*_1_ state in MeCN using M06 XCF and calculated the NTOs associated with the dimer’s first 10 lowest transitions (Figure S15), but we could not find an electronic transition with a charge-transfer (CT) characteristic. However, we did a similar analysis using M11, a functional that has shown great success describing charge-transfer transitions,^83^ and we observed that, in MeCN, the *S*_0_ → *S*_9_ (at 264 nm with *f*_osc_ = 0.0) and *S*_0_ → *S*_10_ (at 263 nm with *f*_osc_ = 0.0) electronic transitions have a CT characteristic associated with the movement of electrons from one chromophore to the other (Figure S16). We could not observe similar electronic transitions in benzene, perhaps because their energy is higher in nonpolar solvents (Figure S17). Experimentally, we observed that the solvation strongly stabilizes the CSS in polar solvents, making it the lowest energy excited state, which TD-DFT fails to capture. To properly describe the CSS *in silico*, we believe that an explicit treatment of the solvent is necessary; the continuum model is clearly not sufficient to explain the role of symmetry breaking. The fluctuations in the local solvent environment around the dimer, which PCM does not model, create an asymmetry in the two sub-units that can drive separation and stabilize the radical cation and anion.^24^

Furthermore, we scanned the *S*_0_ and *S*_1_ PES using DFT/TD-DFT and the M06 functional to explore the flexibility of the ethylene bridge and identify possible structural rearrangements after photoexcitation in polar solvents. Our results suggest that no photoinduced torsional motion occurs about the ethylene bridge (Figure S11).

**Figure 8:**
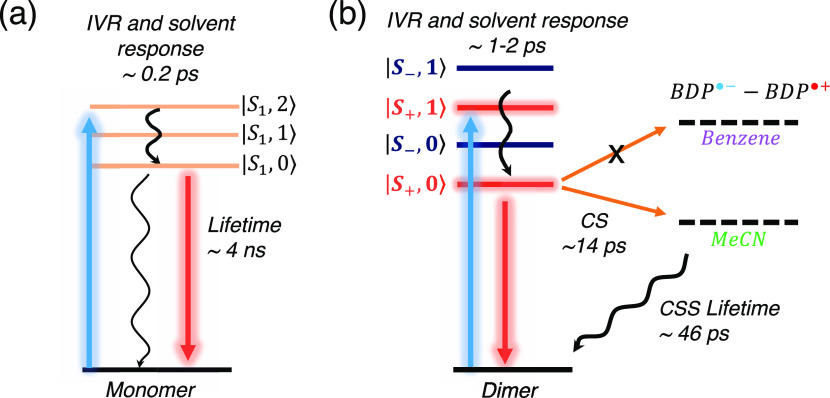
Energy-level diagram of the Bodipy monomer (a) and its dimer (b). Blue and red vertical arrows correspond to absorption and emission processes, respectively, while wavy arrows correspond to non-radiative processes. We show the monomer’s vibronic states, labeled |*S*_1_, *n*⟩, where *n* is the vibrational quantum number. Similarly, we labeled the dimers’ excitonic state as |*S*_±_, *n*⟩, where *S*_+_ and *S*_–_ are the symmetric and antisymmetric linear combination of monomeric *S*_1_ states, respectively.

Finally, Figure 8 summarizes the excited-state dynamics of (a) the monomer and (b) the dimer. Panel (a) presents the dynamics of the monomer regardless of the solvent. We identified two-time constants, the first (<1 ps) is associated with rapid intramolecular vibrational relaxation (IVR) and solvent reorganization and the second (∼4 ns) corresponds to the *S*_1_ lifetime. Panel (b) presents the solvent-dependent dynamics of the dimer after photoexcitation. A 1–2 ps time constant is associated with IVR through the manifold of excitonic-coupled vibrational states. In polar solvents, we observed SB-CS formation in 14 ps due to the strong stabilization of the CSS, which becomes thermodynamically accessible (Δ*G*_CS_ < 0) and leads to a reduced fluorescence quantum yield^20^ and new fsTA absorption bands. Conversely, in nonpolar solvents, the CSS is not accessible (Δ*G*_CS_ > 0), and the 2.1 ns fluorescent lifetime (*S*_1_ lifetime) of the dimer is observed. The CSS energy can be tuned using different solvents, affecting the activation energy and, thus, the kinetics of charge separation and recombination.

## Conclusions

In summary, we determined the solvent-dependent excited-state dynamics of a new α,α-ethylene-bridged Bodipy dimer and studied its excited-state properties using TD-DFT. We evaluated the performance of different XCFs by comparing their ability to reproduce experimental RR spectra and oscillator strengths. The former is a more robust analysis than comparing the experimental and DFT-calculated *E*_00_^Abs^, and we concluded that M06 better describes the excited states of Bodipy derivatives and similar chromophores. Therefore, we have solid foundations to use M06 XCF to interpret the steady-state and time-resolved data. Following this, we determined the monomer’s and dimer’s excited-state dynamics through fsTA and global analysis. The solvent-dependent fsTA measurements showed SB-CS in polar solvents, confirmed with the Rehm–Weller and Marcus electron-transfer calculations. The charge separation is in the Marcus normal regime, and as expected, we observed an increased rate of electron transfer as the polarity of the solvent increased. The charge recombination is in the Marcus inverted regime, where the electron-transfer rate becomes slower for increasing driving force, as observed. TD-DFT only identifies a CSS at very high energies inaccessible from *S*_1_, suggesting that an explicit solvent treatment is required to simulate the environment and stabilize the CSS. Finally, we concluded that photoinduced SB-CS does not lead to a significant structural rearrangement and that fluctuation in the local environment drives and stabilizes the charge separation.

## Supporting Information Available

Complementary steady-state experiments, femtosecond transient absorption data spectra, additional DFT/TDFT calculations results, details of the cyclic voltammetry experiment, experimental DFT/TDF normal mode assignment, and details for the calculations of Marcus parameters (PDF)
