# Supplementary material for: Stimulated Resonance Raman and Excited-State Dynamics in an Excitonically Coupled Bodipy Dimer: A Test for TD-DFT and the Polarizable Continuum Model
Source: J Phys Chem A. 2023 Aug 18;127(34):7156–67. doi: 10.1021/acs.jpca.3c02978 (PMC10476205; doi:10.1021/acs.jpca.3c02978)
Supplement: Supplementary file 1 — jp3c02978_si_001.pdf [file jp3c02978_si_001.pdf]

Supporting Information for:
Stimulated Resonance Raman and Excited State Dynamics in an Excitonically Coupled Bodipy Dimer: A test for TD-DFT and the Polarizable Continuum Model.

Juan S. Sandoval, † Qingbao Gong,†† Lijuan Jiao†† and David W. McCamant.†

†Department of Chemistry, University of Rochester, New York 14627, United States.
††School of Chemistry and Materials Science, Anhui Normal University, Wuhu 241002, China

Table of Contents

Steady-State Experiments **2**
Transition Dipole Moment Orientation **4**
Supplementary Transient Absorption **5**
Molecular Orbitals **6**
TD-DFT Electronic Transition Properties **7**
Cyclic Voltammetry **10**
Calculation of Marcus Parameters **10**
S_0 and S_1 scans along the ethylene bridge using M06 functional **12**
Experimental and DFT/TD-DFT normal mode assignments **13**
Natural Transition Orbitals **16**
References **20**

Steady-State Experiments

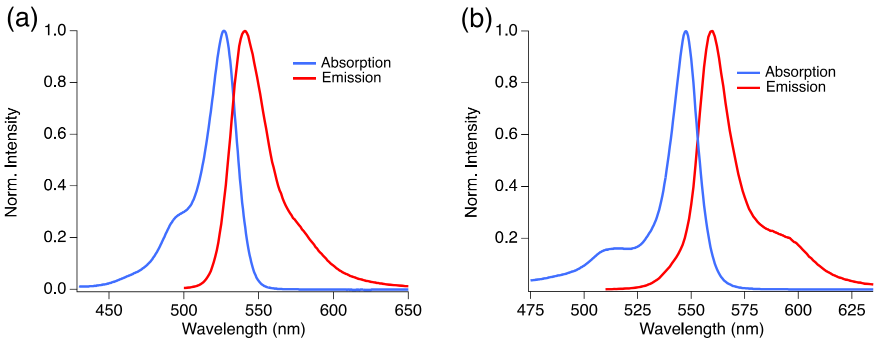

Figure S1. Normalized absorption and emission spectra of the (a) monomer and the (b) dimer.

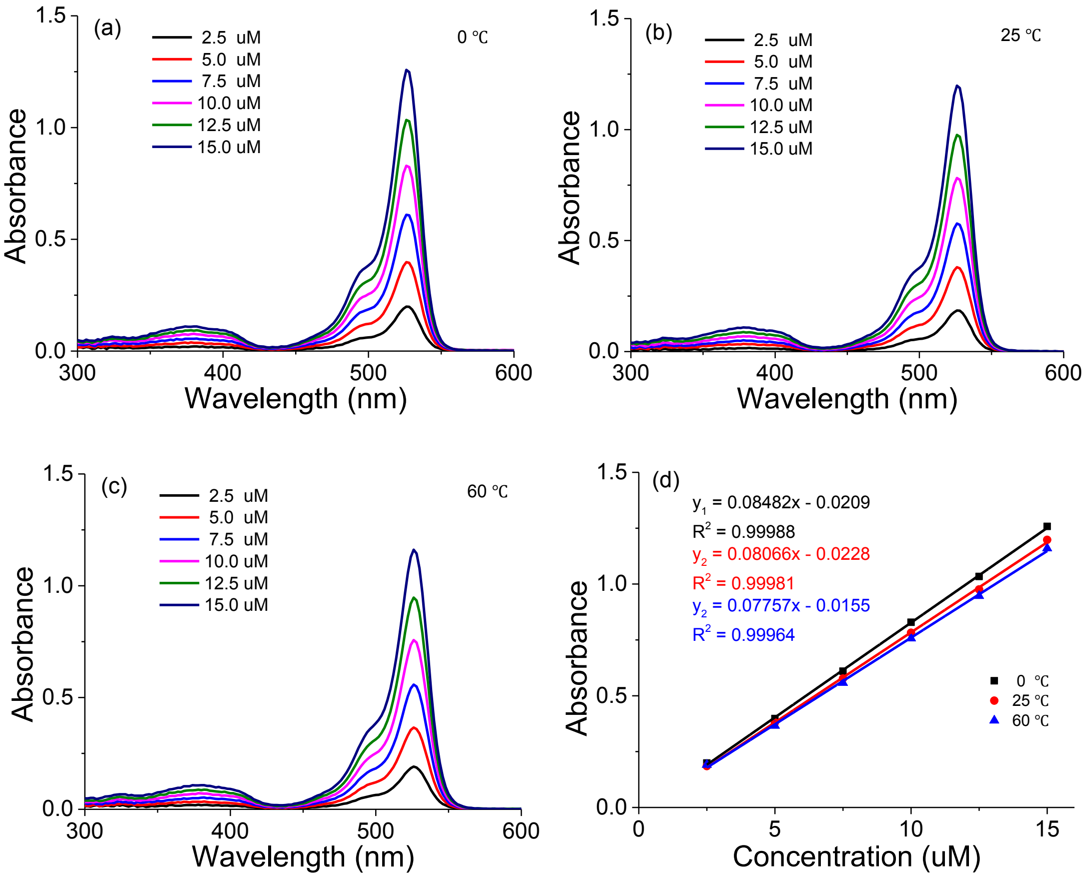

Figure S2. (a, 0 ℃; b, 25 ℃; c, 60 ℃) UV-Vis spectra of the monomer (2.5 - 15.0 μM, benzene) and (d) calibration curves showing the absorbance at λ = 526 nm versus concentration at different temperatures. Molar attenuation coefficients were determined to be (ε_1 = 8.48 x 10^4 L mol^{-1} cm^{-1}, 0 ℃; ε_2 = 8.07 x 10^4 L mol^{-1} cm^{-1}, 25 ℃; ε_3 = 7.76 x 10^4 L mol^{-1} cm^{-1}, 60 ℃).

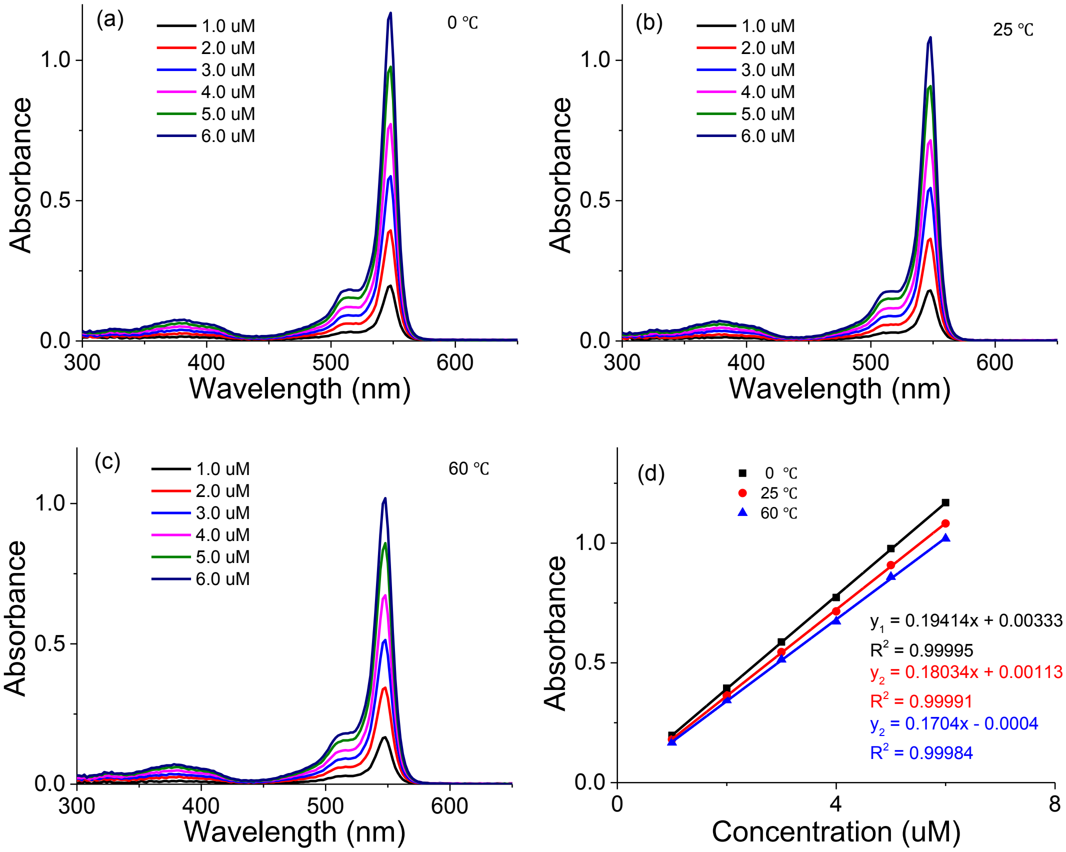

Figure S3. (a, 0 ℃; b, 25 ℃; c, 60 ℃) UV-Vis spectra of the dimer (1.0 - 6.0 μM, benzene) and calibration curves showing the absorbance at λ = 548 nm versus concentration (d). Molar attenuation coefficients were determined to be (ε_1 = 19.4 x 10^4 L mol^{-1} cm^{-1}, 0 ℃; ε_2 = 18.0 x 10^4 L mol^{-1} cm^{-1}, 25 ℃; ε_3 = 17.0 x 10^4 L mol^{-1} cm^{-1}, 60 ℃).

Transition Dipole Moment Orientation

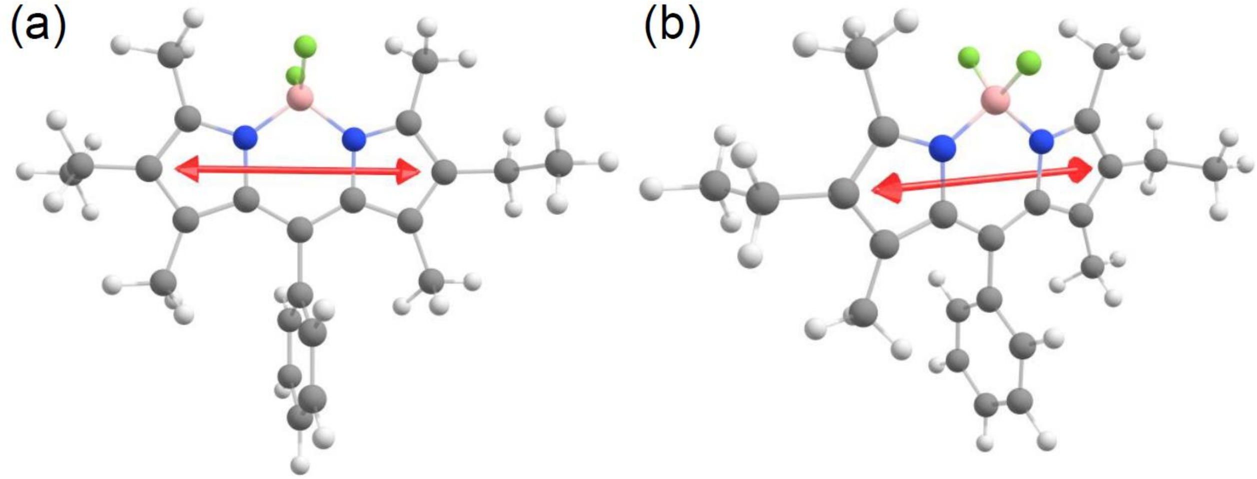

Figure S4. Monomer's transition dipole moment (TDM) vector from two different perspectives using M06 XCF and the ground state optimized geometry. In atomic units, the magnitude of the vector is 3.16, which corresponds to an oscillator strength of 0.696.

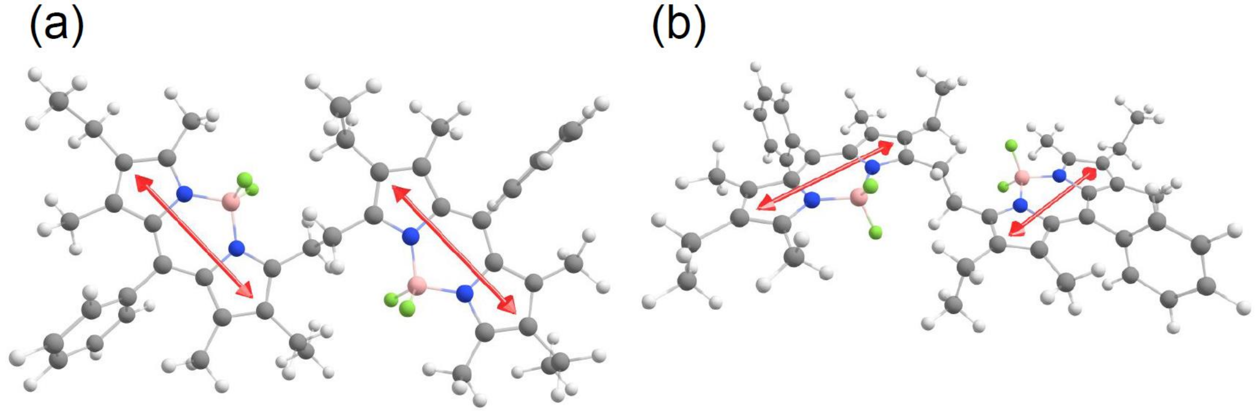

Figure S5. Monomers' TDM vector superimposed on the dimer's optimized ground state geometry from two different perspectives using M06 XCF.

Supplementary Transient Absorption

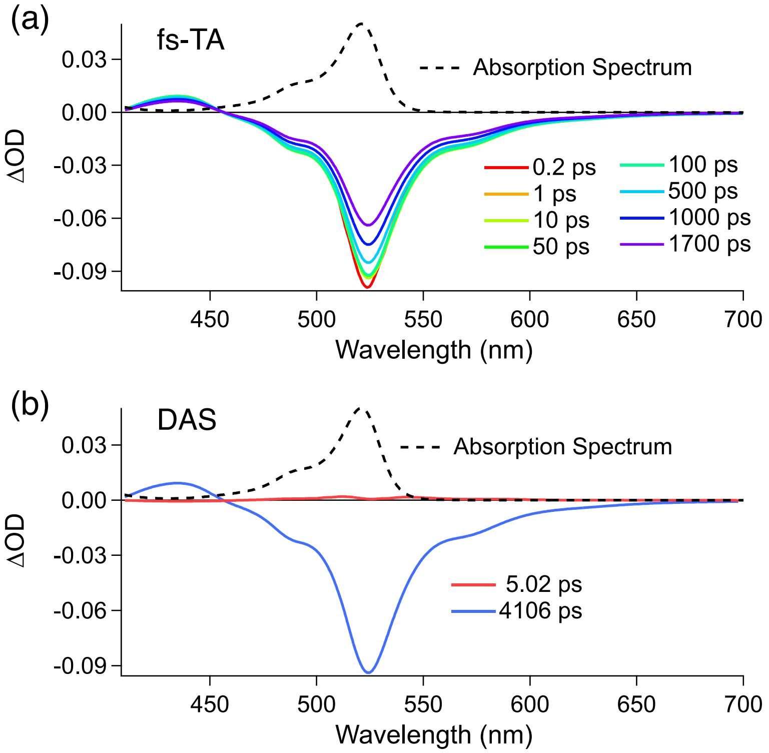

Figure S6. Transient absorption and (b) $DAS(\lambda)$ spectra of the monomer in acetonitrile pumped at 524 nm.

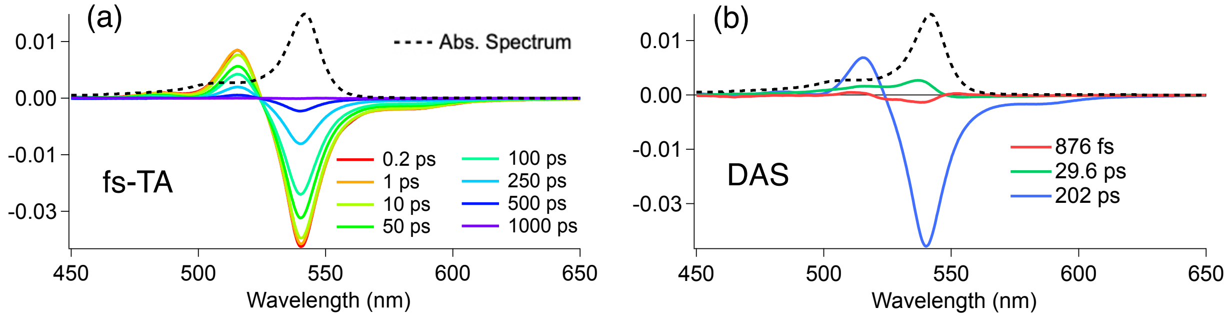

Figure S7. (a) Transient absorption and (b) $DAS(\lambda)$ spectra of the dimer in methanol pumped at 540 nm.

Molecular Orbitals

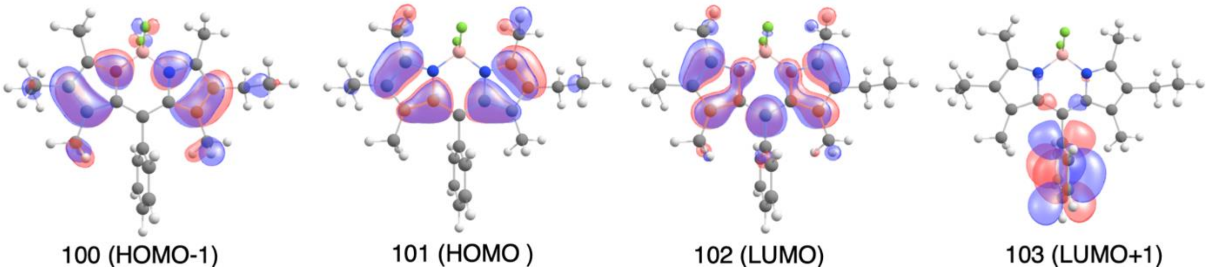

Figure S8. Molecular orbitals for the monomer. Calculated using the M06 exchange-correlation functional at the ground state optimized geometry.

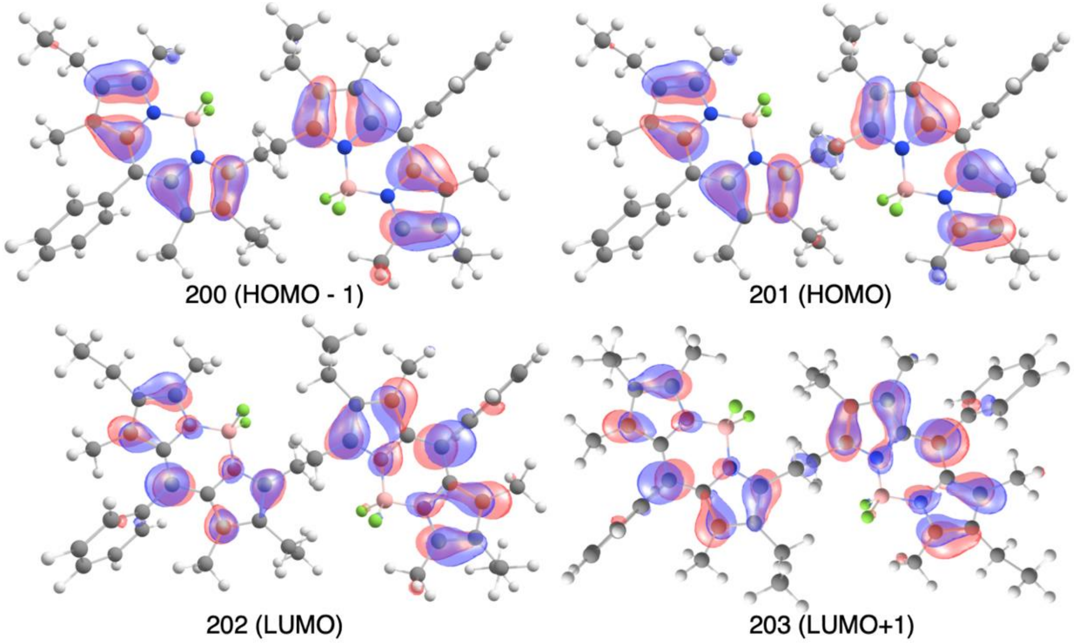

Figure S9. orbitals for the dimer. Calculated using the M06 exchange-correlation functional at the excited state optimized geometry.

TD-DFT Electronic Transition Properties

Table S1. Monomer first five electronic transitions calculated with the labeled functional in benzene using the ground state optimized geometry.

Electronic State/Functional		BHandHLYP		M06		M11	
1	E	23035.1 cm^{-1} (434.1nm)		22148.3 cm^{-1} (451.5nm)		22038.5 cm^{-1} (453.8nm)	
	F_{osc}	0.7782		0.6959		0.7583	
		101→102	0.7014	100→102	0.10024	101→102	0.69768
				101→102	0.70083		
2	E	31583.6 cm^{-1} (316.6nm)		27641.8 cm^{-1} (361.8nm)		32041 cm^{-1} (312.1nm)	
	F_{osc}	0.0838		0.0948		0.0831	
		100→102	0.69973	100→102	0.69813	100→102	0.69645
				101→102	-0.10261		
3	E	33888 cm^{-1} (295.1nm)		29432.5 cm^{-1} (339.8nm)		34270 cm^{-1} (291.8nm)	
	F_{osc}	0.097		0.0716		0.1072	
		99→102	0.69723	99→102	0.70203	99→102	0.68539
4	E	38924.1 cm^{-1} (256.9nm)		32031.7 cm^{-1} (312.2nm)		38915 cm^{-1} (256.97nm)	
	F_{osc}	0.00883		0.0151		0.1403	
		98→102	0.69704	98→102	0.70187	98→102	0.67578
5	E	40904.8 cm^{-1} (244.5nm)		34460.1 cm^{-1} (290.2nm)		43683.4 cm^{-1} (228.9nm)	
	F_{osc}	0.002		0.0015		0.0026	
		97→102	0.70085	97→102	0.70468	97→102	0.51544
						97→103	-0.32041
						98→104	-0.31449
						101→103	0.14282

Table S2. Monomer first five electronic transitions calculated with the M06 functional in benzene using the S_1 optimized geometry.

Electronic State/Functional		M06	
1	E	479.6 nm	
	F_{osc}	0.6189	
		101→102	0.7011
2	E	371.7 nm	
	F_{osc}	0.0983	
		100→102	0.69832
3	E	348.97 nm	
	F_{osc}	0.0823	
		99→102	0.69699
4	E	321.7nm	
	F_{osc}	0.1212	
		98→102	0.69281
5	E	306.3 nm	
	F_{osc}	0.0056	
		101→103	0.6669
		101→105	-0.1612
		101→107	-0.10109

Table S3. Dimer first ten electronic transitions calculated with the labeled functional in benzene using the (left) ground state and (right) S_1 state optimized geometry.

Electronic State/ Functional		BHandHLYP		M06		M11	
1	E	22256.8 cm^{-1} (449.3 nm)		20781.4 cm^{-1} (481.2 nm)		21385.8 cm^{-1} (467.6 nm)	
	F_{osc}	1.5709		1.1532		1.5526	
		200→203	-0.40258	200→203	-0.16213	200→203	0.44479
		201→202	0.57416	201→202	0.68491	201→202	0.53725
2	E	23430.2 cm^{-1}(426.8 nm)		22471.9 cm^{-1} (445.0 nm)		22456.8 cm^{-1} (445.3 nm)	
	F_{osc}	0.0000		0.0000		0.0000	
		200→202	-0.49213	200→202	-0.42748	200→202	0.49220
		201→203	0.49893	201→203	0.55334	201→203	0.49347
3	E	30293.9 cm^{-1} (330.1 nm)		22899 cm^{-1} (436.7 nm)		32000 cm-^{1} (312.5 nm)	
	F_{osc}	0.0000		0.0000		0.1591	
		200→202	0.49675	200→202	0.55634	198→203	-0.47100
		201→203	0.49713	201→203	0.43422	199→202	0.50684
4	E	30515.7 cm^{-}1 (327.7 nm)		23758.6 cm^{-1} (420.9 nm)		32020.5 cm^{-1} (312.3 nm)	
	F_{osc}	0.0292		0.2871		0.0000	
		198→203	-0.13043	200→203	0.67961	198→202	0.50657
		199→202	-0.12898	201→202	0.16891	199→203	-0.47003
		200→203	0.54814				
		201→202	0.40165				
5	E	31525.9 cm^{-1} (317.2 nm)		27548.2 cm^{-1} (363.0 nm)		34258.3 cm^{-1} (291.9 nm)	
	F_{osc}	0.0000		0.1985		0.2731	
		198→202	0.51948	198→203	-0.41957	196→203	-0.46709
		199→203	0.46615	199→202	0.55548	197→203	0.49748
				200→203	-0.10347		
6	E	31565.7 cm^{-1} (316.8 nm)		27578.6 cm^{-1} (362.6 nm)		34317.1 cm^{-1} (291.4 nm)	
	F_{osc}	0.1953		0.0000		0.0000	
		198→203	0.44500	198→202	0.54886	196→202	0.50131
		199→202	0.50866	199→203	-0.42887	197→203	-0.45787
		200→203	0.18010				
7	E	33738.2 cm-^{1} (296.4 nm)		29282.6 cm-^{1} (341.5 nm)		38580.2 cm^{-1} (259.2 nm)	
	F_{osc}	0.2348		0.1777		0.3780	
		196→202	0.52040	196→203	-0.42521	194→203	-0.32347
		197→203	-0.46440	197→202	0.55827	195→202	0.37557
						200→203	-0.36223
						201→202	0.29019
8	E	33795.2 cm^{-1} (295.9 nm)		29385.8 cm^{-1} (340.3 nm)		38580.2 cm^{-1} (259.2 nm)	
	F_{osc}	0.0000		0.0000		0.0000	
		196→203	-0.45977	196→202	0.55931	194→202	-0.30048
		197→202	0.52284	197→203	-0.42181	195→203	0.31264
						200→202	-0.37451
						201→203	0.37876
9	E	37523.5 cm^{-1} (266.5 nm)		31816.7 cm-^{1} (314.3 nm)		39200.3 cm-^{1} (255.1 nm)	
	F_{osc}	0.0229		0.0000		0.0000	
		194→203	0.46494	194→202	0.14164	194→202	0.38624
		195→202	0.52284	195→203	-0.13301	195→203	-0.34424
				196→202	-0.11311	200→202	-0.31451
				197→203	-0.17462	201→203	0.30935
				198→202	0.39958		
				199→203	0.50807		
10	E	37537.5 cm^{-1} (266.4 nm)		31826.9 cm^{-1} (314.2 nm)		39292.7 cm^{-1} (254.5 nm)	
	F_{osc}	0.0000		0.0162		0.0225	
		194→202	0.51708	194→203	0.13112	194→203	-0.32648
		195→203	0.46710	195→202	-0.17768	195→202	0.32266
				196→203	-0.16329	200→203	0.38575
				197→202	-0.12224	201→202	-0.32581
				198→203	0.51092		
				199→202	0.38281		

Electronic State/ Functional		BHandHLYP		M06		M11	
1	E	21579.6 cm^{-1} (463.4 nm)		19658 cm^{-1} (508.7 nm)		20725.4 cm^{-1} (482.5 nm)	
	F_{osc}	1.5246		1.1191		1.4912	
		200→203	0.38499	200→203	-0.13139	200→203	-0.43707
		201→202	0.58617	201→202	0.69229	201→202	0.54235
2	E	22888.5 cm^{-1} (436.9 nm)		21838.8 cm^{-1} (457.9 nm		21853.1 cm^{-1} (457.6 nm)	
	F_{osc}	0.0000		0.0000		0.0000	
		200→202	0.49197	200→202	-0.39599	200→202	-0.49142
		201→203	0.49912	201→203	0.5783	201→203	0.49273
3	E	29629.6 cm^{-1} (337.5 nm)		22202.5 cm-1 (450.4 nm		31695.7 cm^{-1} (315.5 nm)	
	F_{osc}	0.0000		0.0000		0.1560	
		200→202	0.49772	200→202	0.57998	198→203	0.4677
		201→203	-0.49642	201→203	0.40149	199→202	0.51043
4	E	29967 cm^{-1} (333.7 nm)		23523.9 cm^{-1} (425.1 nm		31725.9 cm-^{1} (315.2 nm	
	F_{osc}	0.0466		0.3049		0.0000	
		198→203	0.11589	200→203	0.68554	198→202	0.51013
		199→202	0.11444	201→202	0.1371	199→203	0.46833
		200→203	0.56535				
		201→202	-0.38561				
5	E	31240.2 cm^{-1} (320.1 nm)		27129.7 cm^{-1} (368.6 nm		34036.8 cm^{-1} (293.8 nm)	
	F_{osc}	0.0000		0.173		0.2873	
		198→202	0.52544	198→203	0.39370	196→203	0.46313
		199→203	0.45999	199→202	0.57394	197→202	0.49884
				200→203	0.10558		
6	E	31289.1 cm^{-1} (319.6 nm)		27151.8 cm^{-1} (368.3 nm		34094.8 cm-1 (293.3 nm	
	F_{osc}	0.1888		0.0000		0.0000	
		198→203	0.44189	198→202	0.56921	196→202	0.50309
		199→202	0.51763	199→203	0.40382	197→203	0.45357
		200→203	-0.16319				
7	E	33534.5 cm^{-1} (298.2 nm)		28968.7 cm^{-1} (345.2 nm		37608.1 cm^{-1} (265.9 nm)	
	F_{osc}	0.2411		0.1805		0.2348	
		196→203	0.45700	196→203	0.39767	194→203	0.21017
		197→202	0.52449	197→202	0.57677	195→202	-0.26048
						200→203	0.36833
						200→204	0.18499
						200→211	0.12026
						201→202	0.28742
						201→205	-0.17816
						201→212	0.16677
8	E	33579.6 cm^{-1} (297.8 nm)		29010.7 cm-1 (344.7nm		37622.3 cm^{-1} (265.8 nm)	
	F_{osc}	0.0000		0.0000		0.0000	
		196→202	0.52739	196→202	0.57888	194→202	0.10199
		197→203	0.45163	197→203	0.39112	195→203	-0.12137
						200→202	0.30583
						200→205	-0.20964
						200→212	0.19793
						201→203	0.31241
						201→204	0.29972
						201→207	-0.11646
						201→211	0.18782
9	E	37271.7 cm^{-1} (268.3 nm)		31466.3 cm^{-1} (317.8 nm		37936.3 cm^{-1} (263.6 nm)	
	F_{osc}	0.0580		0.1319		0.0000	
		194→203	0.45792	194→203	-0.40288	194→202	0.22359
		195→202	0.52373	195→202	0.55028	195→203	-0.23441
				198→203	-0.12407	200→202	0.28545
						200→205	0.18816
						200→212	-0.17156
						201→203	0.28532
						201→204	-0.27198
						201→207	0.10810
						201→211	-0.16509
10	E	37299.5 cm^{-1} (268.1 nm)		31476.2 cm^{-1} (317.7 nm		38008.4 cm-^{1} (263.1 nm	
	F_{osc}	0.0000		0.0000		0.1669	
		194→202	0.52068	194→202	-0.34134	194→203	-0.19358
		195→203	0.46105	195→203	0.27720	195→202	0.22493
				197→203	0.13150	200→204	0.29853
				198→202	-0.30556	201→205	-0.28224
				199→203	0.42782	201→212	0.25505

Table S4. Dimer first ten electronic transitions calculated with the labeled functional in acetonitrile using the (left) ground state and (right) S_1 state optimized geometry.

Electronic State/ Functional		BHandHLYP		M06		M11	
1	E	22446.7 cm^{-1} (445.5 nm)		20916.1 cm^{-1} (478.1 nm)		21607.6 cm^{-1} (462.8 nm)	
	F_{osc}	1.4681		1.0697		1.4417	
		200→203	-0.40256	200→203	-0.16108	200→203	-0.44028
		201→202	0.57329	201→202	0.68435	201→202	0.52963
2	E	23596 cm^{-1} (423.8 nm)		22547.9 cm-1 (443.5 nm		22634.7 cm^{-1} (441.8 nm)	
	F_{osc}	0.0000		0.0000		0.0042	
		200→202	-48920	200→202	-0.39762	200→202	-0.48883
		201→203	0.50065	201→203	0.57343	201→203	0.49472
3	E	30385.9 cm^{-1} (329.1 nm)		22977.9 cm^{-1} (435.2 nm)		31847.1 cm^{-1} (314 nm)	
	F_{osc}	0.0000		0.0000		0.0972	
		200→202	0.49873	200→202	0.57703	198→202	0.4417
		201→203	0.49486	201→203	0.4058	198→203	0.38103
						199→202	0.31469
						199→203	0.18805
4	E	30599.8 cm^{-1} (326.8 nm)		23809.5 cm^{-1} (420 nm)		32030.7 cm^{-1} (312.2 nm)	
	F_{osc}	0.029		0.2634		0.0722	
		198→203	0.13901	200→203	0.67882	198→202	0.27806
		199→202	0.13879	201→202	0.16922	198→203	-0.23868
		200→203	0.54314			199→202	-0.37201
		201→202	0.40137			199→203	0.45608
5	E	31515.9 cm^{-1} (317.3 nm)		27548.2 cm^{-1} (363 nm)		34176.3 cm- (292.6 nm)	
	F_{osc}	0.0000		0.2032		0.1899	
		198→202	0.51701	198→203	0.42089	196→202	0.44172
		199→203	0.4657	199→202	0.5527	196→203	0.40846
				200→203	-0.10712	197→202	0.28776
6	E	31565.7 cm^{-1} (316.8 nm)		27586.2 cm^{-1} (362.5 nm)		34352.5 cm^{-1} (291.1 nm)	
	F_{osc}	0.1958		0.0000		0.0936	
		198→203	0.44112	198→202	0.54610	196→202	0.27117
		199→202	0.50307	199→203	0.42984	196→203	-0.14257
		200→203	-0.19304			197→202	-0.36785
						197→203	0.47802
7	E	33783.8 cm^{-1} (296 nm)		29334.1 cm^{-1} (340.9 nm)		37383.2 cm^{-1} (267.5 nm)	
	F_{osc}	0.2404		0.1899		0.1990	
		196→203	-0.46262	196→203	0.42583	194→202	0.31867
		197→202	0.51728	197→202	0.55631	194→203	0.36924
						195→202	0.39506
						195→203	0.16852
						200→203	-0.10468
8	E	33829.5 cm^{-1} (295.6 nm)		29377.2 cm^{-1} (340.4 nm)		37622.3 cm^{-1} (265.8 nm)	
	F_{osc}	0.0000		0.0000		0.0750	
		196→202	0.52027	196→202	0.55887	194→202	0.35937
		197→203	-0.45820	197→203	0.42113	194→203	-0.22414
						195→202	-0.24860
						195→203	0.42895
9	E	36205.6 cm^{-1} (276.2 nm)		30665.4 cm^{-1} (326.1 nm)		38138.8 cm^{-1} (262.2 nm)	
	F_{osc}	0.0155		0.0297		0.0002	
		194→203	-0.46559	194→203	0.44396	200→202	0.14091
		195→202	0.51724	195→202	0.54499	200→204	0.17071
						200→205	0.27246
						200→212	-0.18266
						201→203	0.14222
						201→204	0.42207
						201→205	0.16443
						201→211	0.13737
						201→212	-0.13209
10	E	36218.8 cm^{-1} (276.1 nm)		30684.3 cm^{-1} (325.9 nm)		38402.5 cm^{-1} (260.4 nm)	
	F_{osc}	0.0000		0.0000		0.0002	
		194→202	0.51568	194→202	0.53967	200→203	0.14742
		195→203	-0.46705	195→203	0.44994	200→204	0.35285
						200→205	-0.19707
						200→211	0.14200
						201→202	0.10366
						201→204	-0.16977
						201→205	0.34110
						201→212	-0.19673

Electronic State/ Functional		BHandHLYP		M06		M11	
1	E	21949.1 cm^{-1} (455.6 nm)		20028 cm^{-1} (499.3 nm)		21128.2 cm^{-1} (473.3 nm)	
	F_{osc}	1.4501		1.0471		1.4056	
		200→203	-0.38519	200→203	-0.1223	200→202	-0.10265
		201→202	0.58556	201→202	0.69354	200→203	-0.4296
						201→202	0.53771
2	E	23250.4 cm^{-1} (430.1 nm)		22177.9 cm^{-1} (450.9 nm)		22246.9 cm^{-1} (449.5 nm)	
	F_{osc}	0.0000		0.0000		0.0162	
		200→202	-0.49027	200→202	-0.31965	200→202	-0.4816
		201→203	0.50012	201→203	0.62242	201→203	0.49609
3	E	29815.1 cm^{-1} (335.4 nm)		22416.5 cm^{-1} (446.1 nm)		31605.6 cm^{-1} (316.4 nm)	
	F_{osc}	0.0000		0.0000		0.0902	
		200→202	0.499	200→202	0.62423	198→202	0.47299
		201→203	0.4952	201→203	0.32699	198→203	0.37449
						199→202	0.29419
						199→203	0.17065
4	E	30147.7 cm^{-1} (331.7 nm)		23713.5 cm^{-1} (421.7 nm)		31867.4 cm^{-1} (313.8 nm)	
	F_{osc}	0.0437		0.2970		0.0726	
		198→203	0.12240	200→203	0.68585	198→202	0.24956
		199→202	0.12465	201→202	0.12936	198→203	-0.22922
		200→203	0.56130			199→202	-0.38027
		201→202	0.38597			199→203	0.46968
5	E	31308.7 cm^{-1} (319.4 nm)		27233.1 cm^{-1} (367.2 nm)		34013.6 cm^{-1} (294.0 nm)	
	F_{osc}	0.0000		0.1848		0.1714	
		198→202	0.52318	198→203	-0.39703	195→202	0.10124
		199→203	0.46021	199→202	0.57005	196→202	0.44835
				200→203	-0.11277	196→203	0.37991
						197→202	0.29947
						197→203	0.12678
6	E	31348 cm^{-1} (319 nm)		27270.2 cm^{-1} (366.7 nm)		34246.6 cm^{-1} (292.0 nm)	
	F_{osc}	0.1862		0.0000		0.1057	
		198→203	0.44077	198→202	0.56507	196→202	0.26856
		199→202	0.51247	199→203	-0.40585	196→203	-0.19878
		200→203	-0.17570			197→202	-0.35594
						197→203	0.46440
7	E	33647.4 cm^{-1} (297.2 nm)		29103.6 cm^{-1} (343.6 nm)		36954.9 cm^{-1} (270.6 nm)	
	F_{osc}	0.2352		0.1921		0.2399	
		196→203	-0.45632	196→203	0.39809	194→202	0.27550
		197→202	0.52174	197→202	0.57550	194→203	0.30064
						195→202	0.42141
						195→203	0.22878
						200→203	-0.13210
						201→202	-0.11812
8	E	33692.7 cm^{-1} (296.8 nm)		29146 cm^{-1} (343.1 nm)		37355.2 cm^{-1} (267.7 nm)	
	F_{osc}	0.0000		0.0000		0.1053	
		196→202	0.52541	196→202	0.57833	194→202	0.35914
		197→203	-0.45004	197→203	0.39024	194→203	-0.30567
						195→202	-0.20414
						195→203	0.36789
						197→203	-0.10243
						200→202	-0.10491
						200→203	0.11991
						201→203	-0.12620
9	E	35984.2 cm^{-1} (277.9 nm)		30376.7 cm^{-1} (329.2 nm)		37821.5 cm^{-1} (264.4 nm)	
	F_{osc}	0.0310		0.0733		0.0007	
		194→203	0.45919	194→203	0.42121	200→202	0.22560
		195→202	0.52090	195→202	0.56051	200→204	0.12324
						200→205	0.26472
						200→212	-0.17531
						201→202	0.12582
						201→203	0.21037
						201→204	0.39081
						201→211	0.13105
10	E	35997.1 cm^{-1} (277.8 nm)		30422.9 cm^{-1} (328.7 nm)		38080.7 cm^{-1} (262.6 nm)	
	F_{osc}	0.0000		0.0000		0.0008	
		194→202	0.51912	194→202	0.55321	200→203	0.19386
		195→203	0.46068	195→203	0.42851	200→204	0.33646
						200→205	-0.16372
						201→204	-0.16065
						201→205	0.33972
						201→212	-0.19866

Cyclic Voltammetry

We used a Princeton Applied Research Model 263A potentiostat/galvanostat to control the potential during the experiment. The working and counter electrode were platinum, and the reference electrode was saturated calomel electrode (SCE) (+0.242 V vs NHE) soaked in KCl. We used (TBA)PF_6 as supporting electrolyte. The instrumentation was controlled with PowerSuite 2.60. The sample had a concentration of 1 mM in acetonitrile. The sample was purged with argon for 15 min prior the scan and the experiment was carried out under argon atmosphere. The scan rate was 100mV/s.

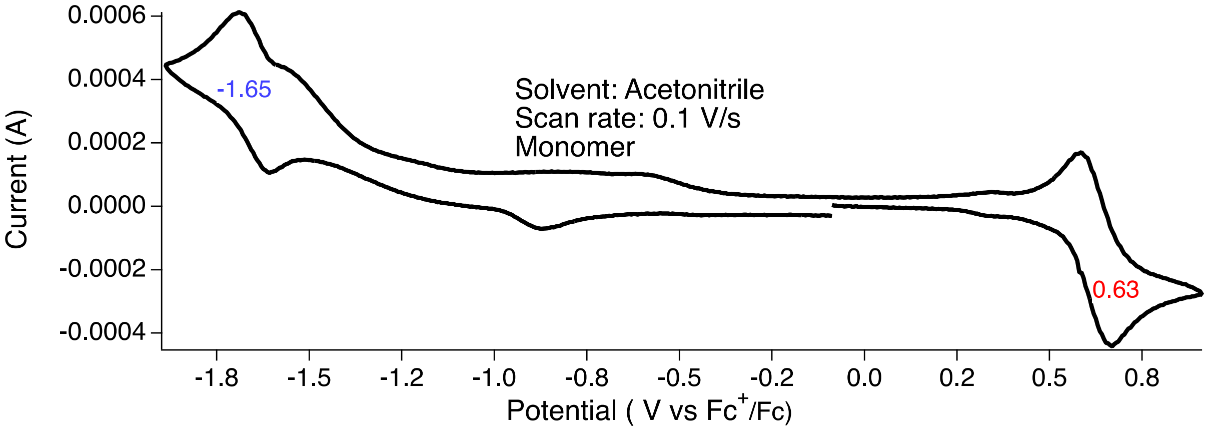

Figure S10. Cyclic voltammogram of the monomer in acetonitrile.

Calculation of Marcus Parameters

The solvent reorganization energy for charge-separation is calculated using the continuum dielectric theory (Nitzan, 2006) as follows:

$$\lambda_s = \frac{e^2}{4\pi\varepsilon_0}\left(\frac{1}{2R_A} + \frac{1}{2R_D} - \frac{1}{R_{DA}}\right)\left(\frac{1}{\varepsilon_{op}} - \frac{1}{\varepsilon_s}\right),$$

where the radii of the donor (R_A) and acceptor (R_A) are taken as half of donor-acceptor distance (R_{DA}). ε_{op} is the optical response and ε_s the dielectric constant (static response) of the solvent.

The inner sphere reorganization energy is calculated using the four-point method (Wu & Van Voorhis, 2006):

$$\lambda_i^{4p} = \left[E_{D^+}\left(R_{eq}^{D^*}\right) - E_{D^+}\left(R_{eq}^{D^+}\right)\right] + \left[E_{A^-}\left(R_{eq}^{G}\right) - E_{A^-}\left(R_{eq}^{A^-}\right)\right],$$

Where $R_{eq}^{D^*}$ and $R_{eq}^{D^+}$ are the equilibrium geometries of the excited and cationic states, respectively. Similarly, R_{eq}^{G} and $R_{eq}^{A^-}$ are the equilibrium geometries of the ground and anionic states, respectively. The level of theory was M06 / 6-311G* in acetonitrile using the polarizable continuum model. The geometry convergence was set to default and the integration grid was set to ultra-fine.

The total reorganization energy for the charge-separation is given by the sum of internal and solvent reorganization energies:

$$\lambda_T = \lambda_s + \lambda_i^{4p}$$

The energy of the Ion-pair (E_{IP}) is calculated via the Rehm-Weller equation (Weller, 1982)

$$E_{IP} = e\big(E_{ox}(D) - E_{red}(A)\big) - k_e \frac{e^2}{\varepsilon_r R_{DA}} + k_e e^2 \left(\frac{1}{R_A} + \frac{1}{R_D}\right)\left(\frac{1}{\varepsilon_r} + \frac{1}{\varepsilon_{MeCN}}\right),$$

Where $E_{ox}(D) - E_{red}(A)$, are the oxidation and reduction potential for the donor (D) and acceptor (A), both being the monomer in this case. ε_{MeCN} the dielectric constant of acetonitrile and ε_s the dielectric constant for another solvent (Methanol or benzene). The free energy gap (ΔG_{CS}) for charge-separation can then be estimated as follows:

$$\Delta G_{CS} = E_{IP} - E_{00}(D),$$

where E_{00} is the “0-0” energy gap, calculated as the crossing point between the normalized absorption and emission spectra.

The electronic coupling is calculated from the classical expression of Marcus electron transfer theory (Marcus, 1956),

$$k = \frac{2\pi}{\hbar} \frac{V_{DA}^2}{\sqrt{4\pi\lambda_T k_B T}} e^{-\frac{(\Delta G_{CS}+\lambda_T)^2}{4\lambda_T k_B T}}.$$

Here, V_{DA} is the donor-acceptor electronic coupling, k_B is the Boltzman constant, T the temperature and k is the rate constant for charge-separation. The rate constant can be related to the charge-transfer time constant ($k = 1/\tau_{CS}$), obtained from fsTA and global analysis, and so we can calculate V_{DA} from the Marcus equation with all other parameters defined by DFT and fsTA.

S_0 and S_1 scans along the ethylene bridge using M06 functional

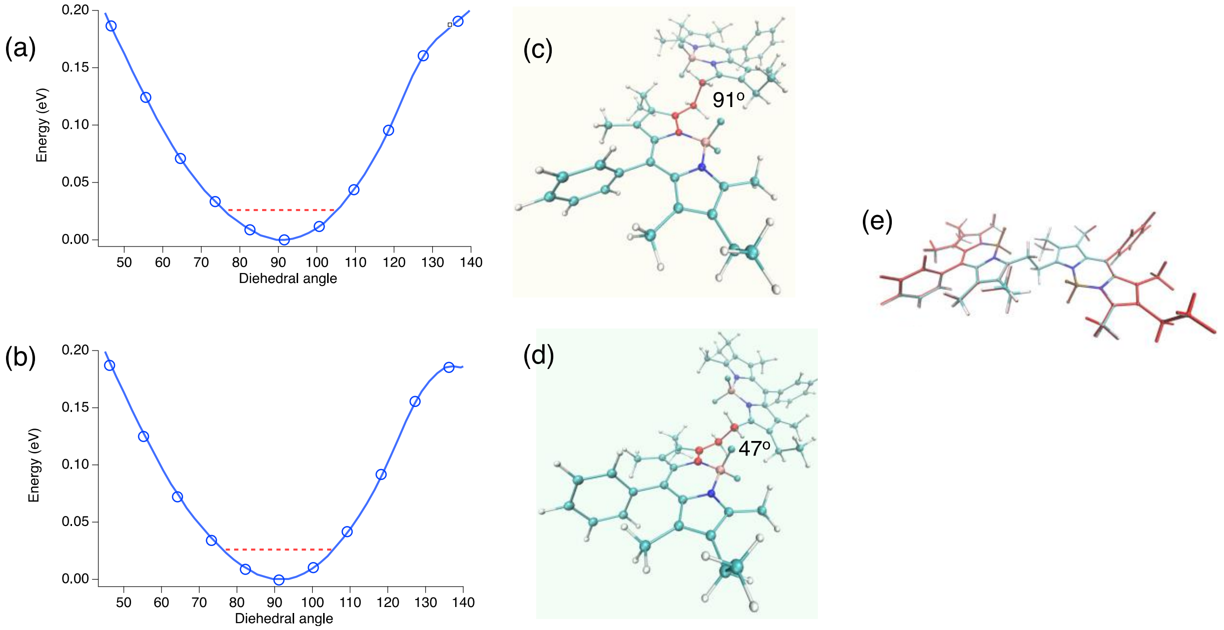

Figure S11. Relaxed potential energy scan on the ethylene bridge for the ground (a) and excited (b) electronic state of the Bodipy dimer. The dashed line in (a) and (b) correspond to the thermal energy ($k_B T$). Two structures at different dihedral angles are presented in (c) and (d). The atoms included in the scan are shown in red in (c) and (d). In (e) we present the superimposed ground and (red) excited state optimized geometry. Only minor changes are observed.

Experimental and DFT/TD-DFT normal mode assignments

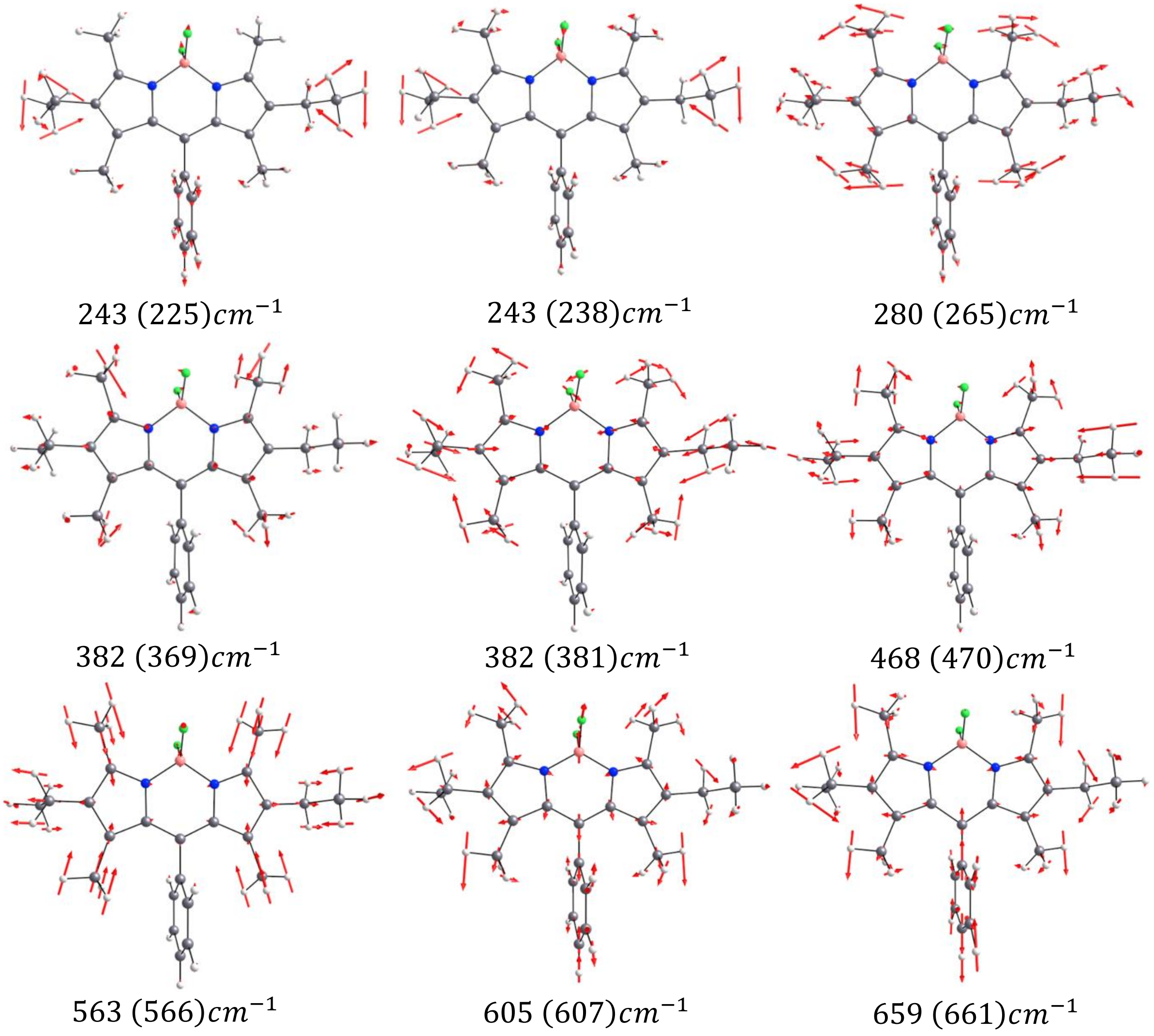

Figure S12. Raman active modes of the Bodipy monomer identified from the experimental Resonance Raman. The experimental frequency is presented, and in parenthesis the DFT (M06/6311G*) associated mode.

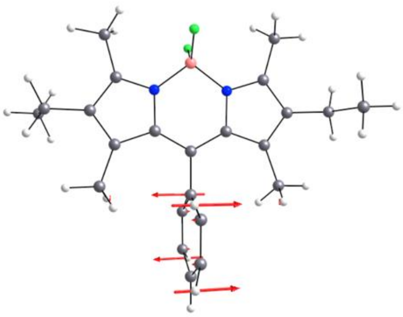

836 (849)cm^{-1}

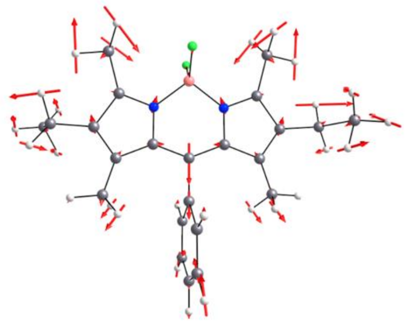

957 (973)cm^{-1}

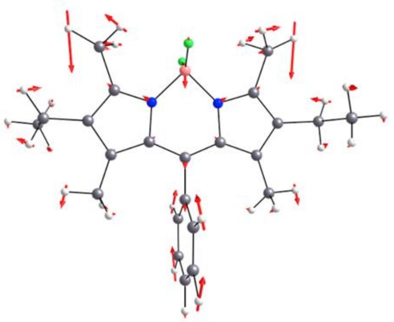

1044 (1073)cm^{-1}

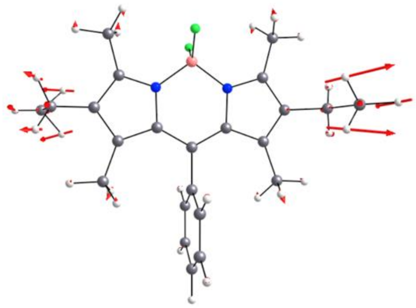

1044 (1085)cm^{-1}

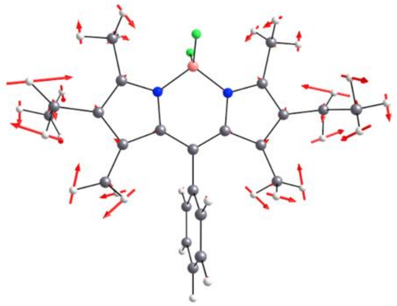

1109 (1116)cm^{-1}

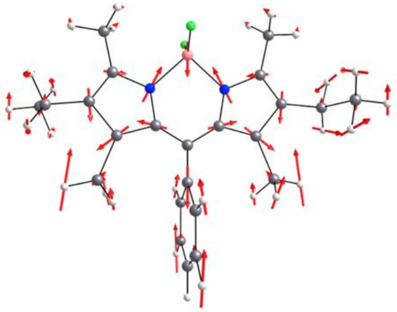

1176 (1207)cm^{-1}

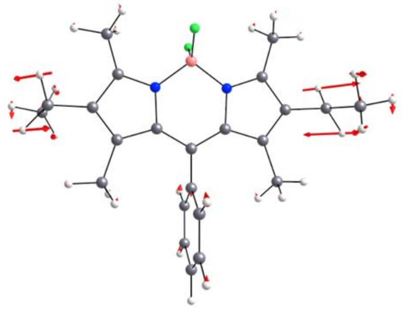

1257 (1281)cm^{-1}

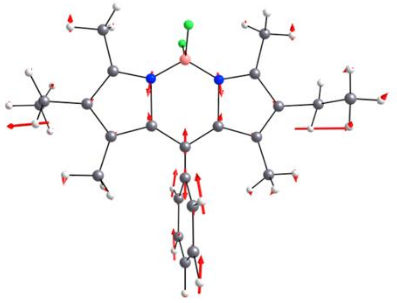

1276 (1304)cm^{-1}

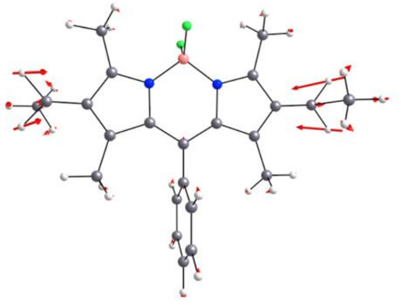

1323 (1340)cm^{-1}

Figure S12. (Continuation) Raman active modes of the Bodipy monomer identified from the experimental Resonance Raman. The experimental frequency is presented and in parenthesis the DFT (M06/6311G*) associated mode.

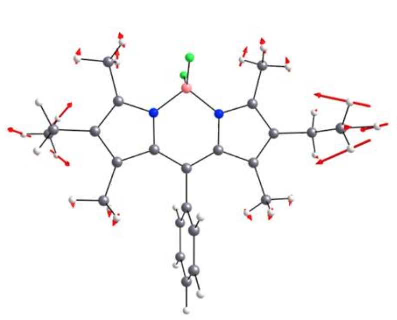

1372 (1393) cm^{-1}

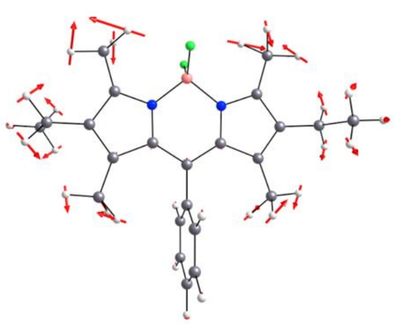

1414 (1456) cm^{-1}

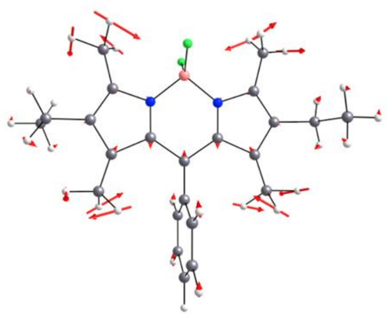

1414 (1460) cm^{-1}

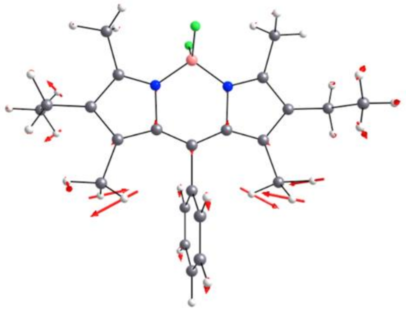

1414 (1467) cm^{-1}

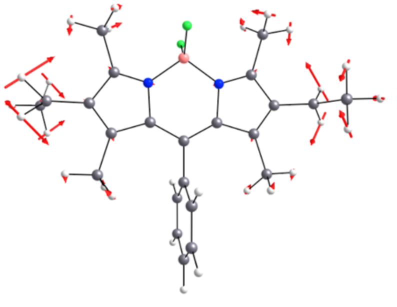

1460 (1489) cm^{-1}

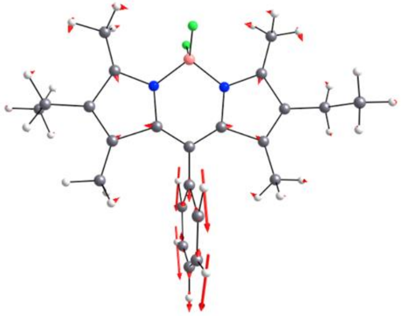

1503 (1521) cm^{-1}

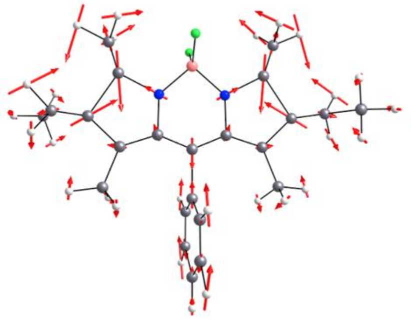

1503 (1544) cm^{-1}

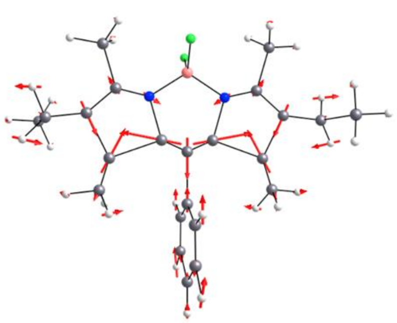

1503 (1563) cm^{-1}

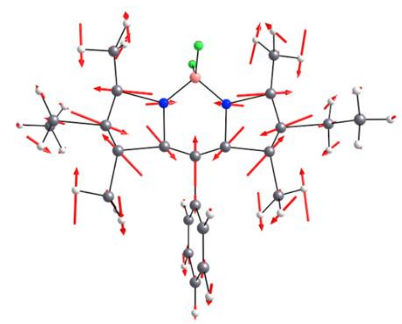

1552 (1599) cm^{-1}

Figure S12. (Continuation) Raman active modes of the Bodipy monomer identified from the experimental Resonance Raman. The experimental frequency is presented and in parenthesis the DFT (M06/6311G*) associated mode.

Natural Transition Orbitals

$(a)\ S_0 \rightarrow S_1$
$f = 0.70$
$\lambda = 451\ nm$

$(b)\ S_0 \rightarrow S_2$
$f = 0.1$
$\lambda = 362\ nm$

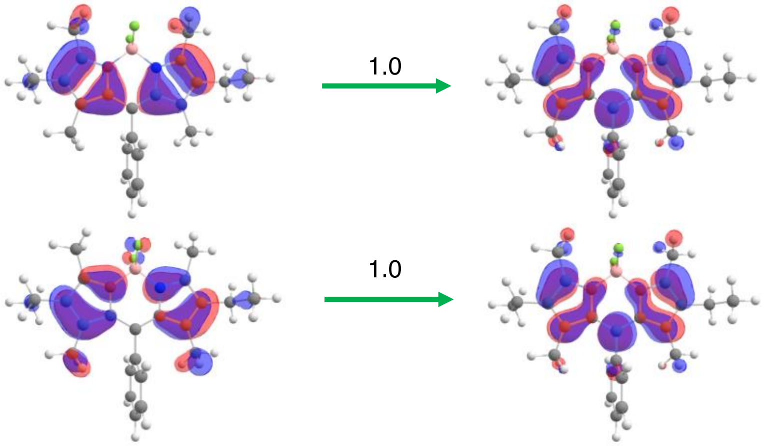

Figure S13: Natural transition orbitals of the first two electronic transitions of the Bodipy monomer in benzene using the ground state optimized geometry and the M06 XCF. The oscillator strength (f), excitation energy (λ), and the associated eigenvalue are presented for each transition.

$(a)\ S_0 \rightarrow S_1$
$f = 0.62$
$\lambda = 480\ nm$

$(b)\ S_0 \rightarrow S_2$
$f = 0.1$
$\lambda = 372\ nm$

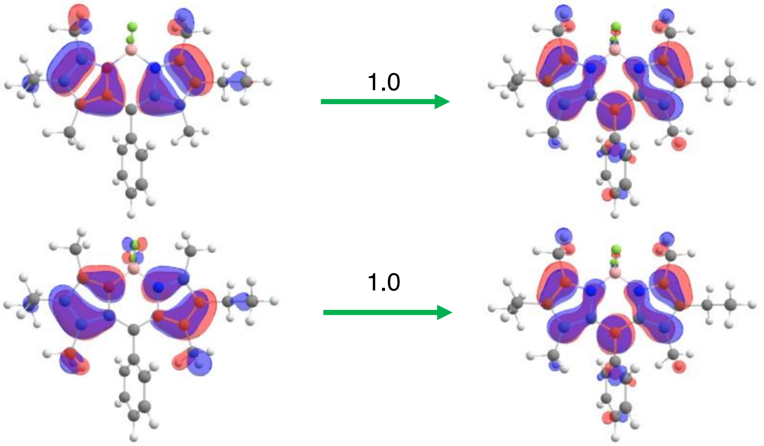

Figure S14: Natural transition orbitals of the first two electronic transitions of the Bodipy monomer in benzene using the excited state optimized geometry and the M06 XCF. The oscillator strength (f), excitation energy (λ), and the associated eigenvalue are presented for each transition.

$(a)\ S_0 \rightarrow S_1$
$f = 1.05$
$\lambda = 499\ nm$

$(b)\ S_0 \rightarrow S_2$
$f = 0.00$
$\lambda = 451\ nm$

$(c)\ S_0 \rightarrow S_3$
$f = 0.00$
$\lambda = 446\ nm$

$(d)\ S_0 \rightarrow S_4$
$f = 0.30$
$\lambda = 421.7\ nm$

$(e)\ S_0 \rightarrow S_5$
$f = 0.19$
$\lambda = 367\ nm$

$(f)\ S_0 \rightarrow S_6$
$f = 0.00$
$\lambda = 366\ nm$

$(g)\ S_0 \rightarrow S_7$
$f = 0.19$
$\lambda = 344\ nm$

$(h)\ S_0 \rightarrow S_8$
$f = 0.00$
$\lambda = 343\ nm$

$(i)\ S_0 \rightarrow S_9$
$f = 0.07$
$\lambda = 329\ nm$

$(j)\ S_0 \rightarrow S_{10}$
$f = 0.00$
$\lambda = 329\ nm$

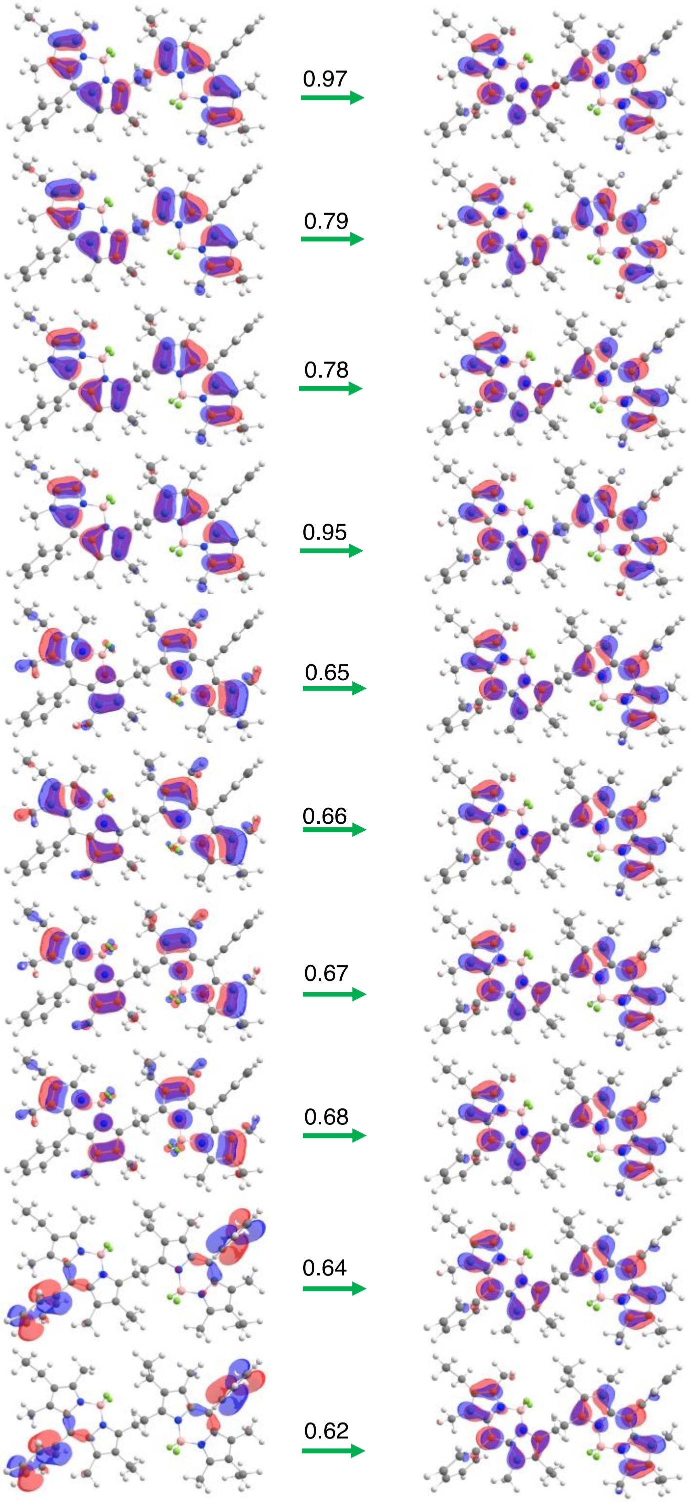

Figure S15: Natural transition orbitals of the first ten electronic transitions of the Bodipy dimer in **MeCN** using the **S_1** state-optimized geometry and the **M06** XCF. The oscillator strength (f), excitation energy (λ), and the associated eigenvalue are presented for each transition.

$(a)\ S_0 \rightarrow S_1$ $f = 1.41$ $\lambda = 473\ nm$

$(b)\ S_0 \rightarrow S_2$ $f = 0.016$ $\lambda = 449\ nm$

$(c)\ S_0 \rightarrow S_3$ $f = 0.09$ $\lambda = 316\ nm$

$(d)\ S_0 \rightarrow S_4$ $f = 0.07$ $\lambda = 314\ nm$

$(e)\ S_0 \rightarrow S_5$ $f = 0.17$ $\lambda = 294\ nm$

$(f)\ S_0 \rightarrow S_6$ $f = 0.11$ $\lambda = 292\ nm$

$(g)\ S_0 \rightarrow S_7$ $f = 0.24$ $\lambda = 271\ nm$

$(h)\ S_0 \rightarrow S_8$ $f = 0.11$ $\lambda = 268\ nm$

$(i)\ S_0 \rightarrow S_9$ $f = 0.00$ $\lambda = 264\ nm$

$(j)\ S_0 \rightarrow S_{10}$ $f = 0.00$ $\lambda = 263\ nm$

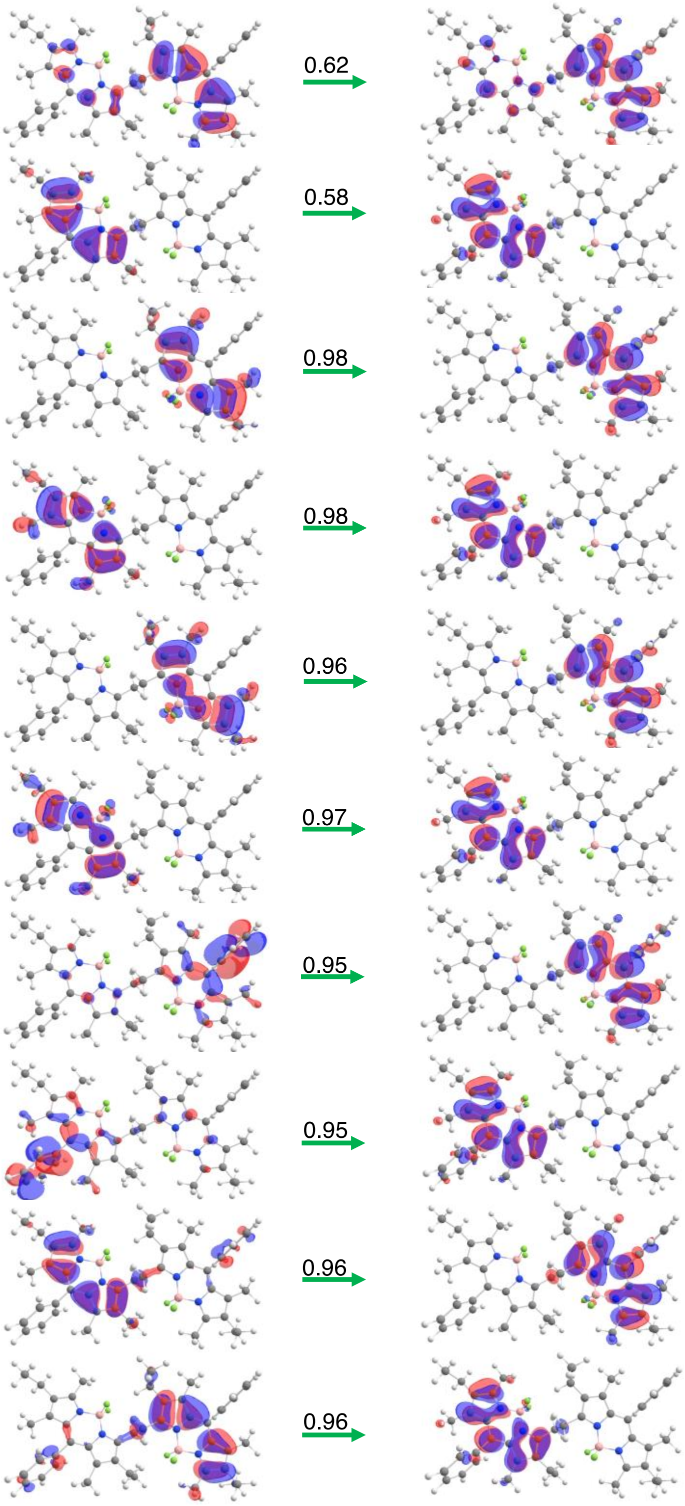

Figure S16: Natural transition orbitals of the first ten electronic transitions of the Bodipy dimer in **MeCN** using the $\mathbf{S_1}$ state-optimized geometry and the **M11** XCF. The oscillator strength (f), excitation energy (λ), and the associated eigenvalue are presented for each transition.

$(a)\ S_0 \rightarrow S_1$
$f = 1.49$
$\lambda = 482\ nm$

$(b)\ S_0 \rightarrow S_2$
$f = 0.00$
$\lambda = 458\ nm$

$(c)\ S_0 \rightarrow S_3$
$f = 0.16$
$\lambda = 316\ nm$

$(d)\ S_0 \rightarrow S_4$
$f = 0.00$
$\lambda = 316\ nm$

$(e)\ S_0 \rightarrow S_5$
$f = 0.29$
$\lambda = 294\ nm$

$(f)\ S_0 \rightarrow S_6$
$f = 0.00$
$\lambda = 293\ nm$

$(g)\ S_0 \rightarrow S_7$
$f = 0.23$
$\lambda = 266\ nm$

$(h)\ S_0 \rightarrow S_8$
$f = 0.00$
$\lambda = 266\ nm$

$(i)\ S_0 \rightarrow S_9$
$f = 0.00$
$\lambda = 264\ nm$

$(j)\ S_0 \rightarrow S_{10}$
$f = 0.17$
$\lambda = 263\ nm$

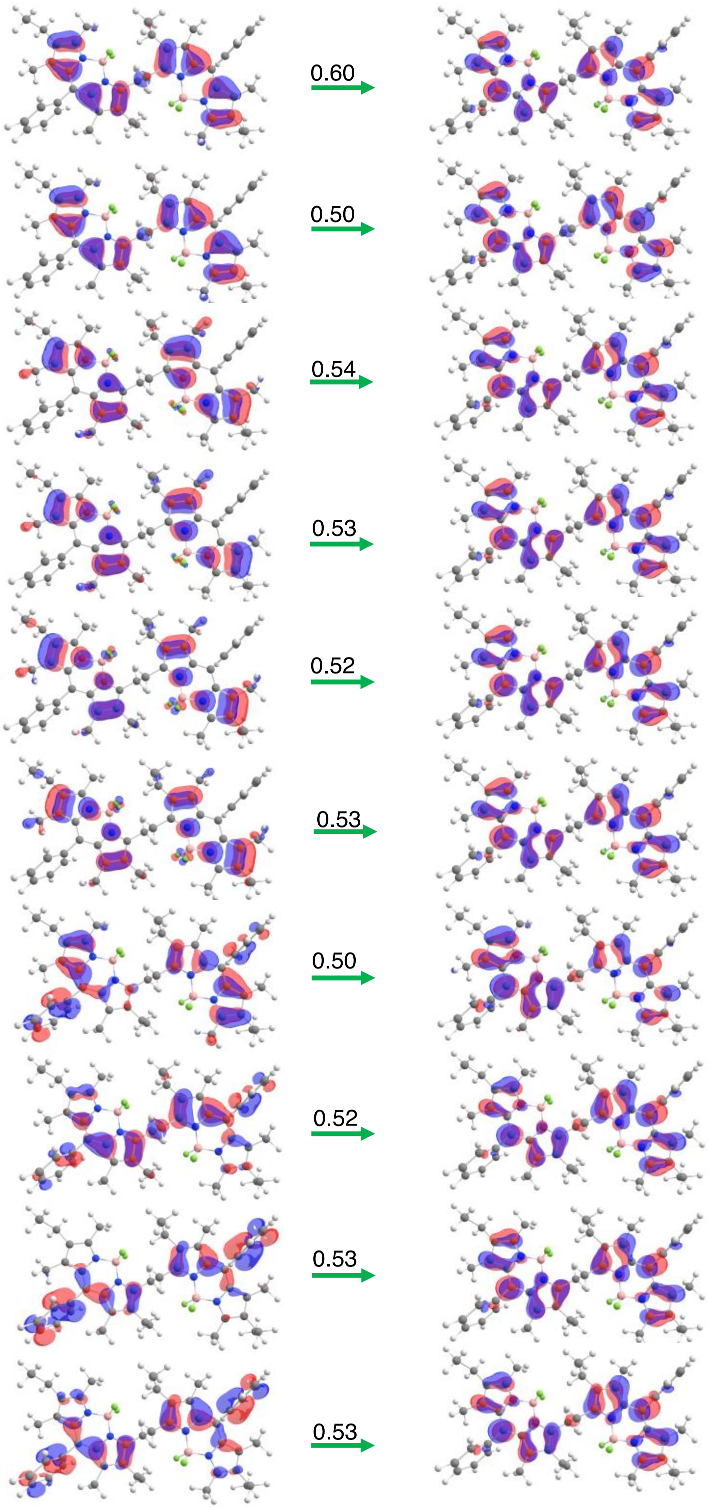

Figure S17: Natural transition orbitals of the first ten electronic transitions of the Bodipy dimer in **benzene** using the **S_1** state-optimized geometry and the **M11** XCF. The oscillator strength (f), excitation energy (λ), and the associated eigenvalue are presented for each transition.

References

Marcus, R. A. (1956). On the Theory of Oxidation-Reduction Involving Electron Transfer I. *The Journal of Chemical Physics* , 966-978.

Nitzan, A. (2006). *Chemical Dynamics in Condense Phases.* Oxford University Press.

Weller, A. (1982). Photoinduced Electron Transfer in Solution: Exciplex and Radical Ion Pair Formation Free Enthalpies and their Solvent Dependence. *Zeitschrift für Physikalische Chemie*, 93-98.

Wu, Q., & Van Voorhis, T. (2006). Direct Calulcation of Electron Transfer Parameters trough Constrained Density Functional Theory. *The Journal of Physical Chemistry A*, 9212-9218.
